# Effectiveness of a community-level social mobilization intervention in achieving the outcomes of polio vaccination campaigns during the post-polio-endemic period: Evidence from CORE Group polio project in Uttar Pradesh, India

**DOI:** 10.1186/s12889-021-11425-0

**Published:** 2021-07-10

**Authors:** Manojkumar Choudhary, Roma Solomon, Jitendra Awale, Rina Dey, Jagajeet Prasad Singh, William Weiss

**Affiliations:** 1 CORE Group Polio Project, India, 303, Bestech Chambers, B-Block, Sushant Lok-1, Gurgaon, Haryana 122002 India; 2 Indian Institute of Health Management Research (IIHMR) University, Jaipur, Rajasthan 302020 India; 3 Department of International Health, Johns Hopkins Bloomberg School of Public Health, Baltimore, MD USA

**Keywords:** Polio, Vaccination campaigns, Supplementary immunization activities, Social mobilization, CORE Group polio project

## Abstract

**Background:**

A social mobilization (SM) initiative contributed to India’s success in polio elimination. This was the CORE Group Polio Project (CGPP) India, a partner of the Uttar Pradesh (UP) SM Network and which continued its SM activities, even during the polio-free period through a network of multi-level social mobilizers. This paper assesses the effects of this community-level SM (CLSM) intervention on the extent of community engagement and performance of polio Supplementary Immunization Activity campaigns (SIAs) during the post-polio-endemic period (i.e., from March 2012 to September 2017).

**Methods:**

This study followed a quasi-experimental design. We used secondary, cluster-level data from CGPP India’s Management Information System, including 52 SIAs held from January 2008 to September 2017, covering 56 blocks from 12 districts of UP. We computed various indicators and performed Generalized Estimating Equations based analysis to assess the statistical significance of differences between the outcomes of intervention and non-intervention areas. We then estimated the effects of the SM intervention using Interrupted time-series, Difference-in-Differences and Synthetic Control Methods. Finally, we estimated the population influenced by the intervention.

**Results:**

The performance of polio SIAs changed over time, with the intervention areas having better outcomes than non-intervention areas. The absence of CLSM intervention during the post-polio-endemic period would have negatively impacted the outcomes of polio SIAs. The percentage of children vaccinated at polio SIA booths, percentage of ‘X’ houses (i.e., households with unvaccinated children or households with out-of-home/out-of-village children or locked households) converted to ‘P’ (i.e., households with all vaccinated children or households without children eligible for vaccination), and percentage of resistant houses converted to polio acceptors would have gone down by 14.1 (Range: 12.7 to 15.5), 6.3 (Range: 5.2 to 7.3) and 7.4 percentage points, respectively. Community engagement would have reduced by 7.2 (Range: 6.6 to 7.7) percentage points.

**Conclusions:**

The absence of CLSM intervention would have significantly decreased the level of community engagement and negatively impacted the performance of polio SIAs of the post-polio-endemic period. The study provides evidence of an added value of deploying additional human resource dedicated to social mobilization to achieve desired vaccination outcomes in hard-to-reach or programmatically challenging areas.

**Supplementary Information:**

The online version contains supplementary material available at 10.1186/s12889-021-11425-0.

## Background

The world is on the verge of eradicating poliomyelitis (polio), and the disease remains endemic only in two countries, namely Afganistan and Pakistan [1]. Out of three, two strains of wild poliovirus have been globally eradicated [2]. The total number of wild polio cases has drastically reduced globally from an estimated 350,000 cases in 1988 [3] to 140 cases in 2020 [4]. Since its inception in 1998, the Global Polio Eradication Initiative (GPEI) created a significant infrastructure for disease surveillance, social mobilization (SM), and vaccine delivery; developed in-depth knowledge and expertise; and learned valuable lessons on reaching the most vulnerable and hard-to-reach populations on earth [5].

India had the highest incidence of wild poliovirus (WPV) during the nineties and was perceived as a challenging and to be the last country to stop polio transmission [6–8]. India followed the GPEI recommendations and applied different strategies to interrupt transmission, such as 1) High levels of routine immunization, 2) High-quality supplementary immunization activities (SIAs), and 3) Sensitive surveillance to identify areas of wild poliovirus transmission and to guide immunization activities [9]. The concentrated efforts of the Government of India and polio partners led to polio elimination, and the country was officially declared polio-free in March 2014. India’s national polio eradication program averted millions of paralytic polio cases, hundred of thousands of polio deaths during the polio-endemic period and economically benefited the country [10].

The social mobilization (SM) initiative was one of the contributors to India’s success in polio elimination [8, 11, 12]. In Uttar Pradesh (UP), India, the polio Social Mobilization Network (SM Net) was established in 2003 to counter the misconceptions and community refusals against the oral polio vaccine [8]. Its partners included UNICEF, CORE Group Polio Project (CGPP), Rotary, and the Indian Government’s and WHO’s National Polio Surveillance Project (NPSP) [13]. The UP SM Net implemented synchronized social mobilization activities using community-level workers called Community Mobilization Coordinators (CMCs), who were supervised by Block Mobilization Coordinators (BMCs), in turn, supervised by District Mobilization Coordinators (DMCs). Initially, the SM Net was focused on vaccine resistance and generating demand for it. However, the network extended its messaging and SM activities over the years to promote and increase routine immunization coverage [13, 14]. The SM Net supported polio eradication in high-risk areas for polio, working with underserved communities to execute SM and other immunization-related activities. Its SM activities were implemented consistently across the CGPP and UNICEF areas [15].

The CMCs - community-level mobilizers of SM Net were deployed to advocate for vaccination in the selected polio high-risk areas (i.e., villages/urban wards) within administrative blocks of a district, designated as ‘CMC areas’. Areas without CMC deployment were designated as ‘non-CMC areas’. Hence, a block or polio-planning unit^1^ included both CMC and non-CMC areas. The CMCs were mostly deployed in the polio High-Risk Areas (HRAs)^2^ because these areas experienced more resistance to polio vaccination and included a more hard-to-reach population than non-CMC areas. Since March 2014, UNICEF gradually withdrew its CMCs, but CGPP continued its community-level social mobilization (CLSM) efforts in the selected 12 districts of UP, India.

A few published studies found that the SM interventions have contributed to the desired outcomes of polio Supplementary Immunization Activity campaigns (SIAs) during India’s polio-endemic period [8, 11, 12, 15]. However, we could not find a study in the peer-reviewed literature that precisely attributed a community-level SM intervention to the programmatic achievements, particularly during the post-polio-endemic era (i.e., after February 2012). Most previous studies that assessed the contribution of Social and Behavior Change Communication (SBCC) or SM intervention in improving vaccination performance had limitations around study design and the availability of data needed to assess the warranted estimates. Our earlier analysis, measuring the magnitude of community engagement in polio SIAs held from 2008 to 2017, found that the CGPP India’s SM intervention led to a significant increase in the CMC areas’ engaged communities [16]. Using the case of CGPP India’s SM Net, this paper estimates the extent of CLSM intervention’s effects on the performance of polio SIAs during the post-polio-endemic period *(*i.e.*, from March 2012 to September 2017)*. We hypothesize that the absence of CGPP India’s CLSM intervention would have adversely affected the polio SIAs outcomes in the CMC areas during the post-polio-endemic period. Information on value addition by the CMCs during the post-polio-endemic era would support policymakers/program managers of other public health programs to think and rationalize deploying additional human resources dedicated to social mobilization.

## Methods

*SM Net intervention during SIAs***–** Polio SIA operation in UP is almost uniform across the districts and includes two main types: (1) Fixed-site or booth-based vaccination^3^; and (2) House-to-house vaccination. Polio SIAs, generally begin on a Sunday with fixed polio vaccination booths for one day. Then the house-to-house vaccination phase begins. The SM Net functionaries in CMC areas engage communities for each polio SIA. CMCs in their areas perform various awareness generation and trust-building activities before each SIA, such as the following: (a) interpersonal (one-to-one and one-to-group) communication with caregivers and family members of children eligible for SIA vaccination; (b) meetings with local influencers; (c) children’s rallies. In addition, DMCs and BMCs help the government prepare for SIAs by developing micro-plans and ensuring the availability of necessary logistics and supplies. On the booth day, the CMCs involve school children encouraging the community to bring the children younger than five years to booths for vaccination. During the house-to-house vaccination, CMCs accompany vaccinators who vaccinate eligible children. If the vaccination team encounters refusal, CMCs engage the local influencers to convince resistant families to allow their children for polio vaccination. After an SIA, the SM Net functionaries visit all the houses with unvaccinated children and encourage family members to go for polio vaccination in the upcoming/next SIA [8, 17, 18].

This study followed a quasi-experimental design that included time-series data with a non-equivalent comparison group. For this study, we defined CMC areas as ‘CLSM intervention areas’, and the areas without CMC deployment were considered ‘Non-intervention areas’ of a block or polio-planning unit.

### Data source

We performed a secondary analysis of data routinely collected through the project Management Information System (MIS) of CGPP India (Refer to Weiss et al., 2011 Choudhary et al., 2019 for more details). The CGPP MIS provided information about various activities and results surrounding each polio SIA such as the following: (a) number of eligible children; (b) number of children vaccinated at SIA booths (i.e., fixed-site vaccination); (c) number of households visited by house-to-house vaccination teams; (d) number of households with all children in household vaccinated during the SIA; (e) number of households with at least one unvaccinated child; and, (f) number of households that refused vaccination, etc. We created a single database from separate data sheets (i.e., monthly progress reports of CGPP India MIS).

### SIAs and analysis period

The study included 52 polio SIAs held from January 2008 to September 2017 in 56 blocks/polio planning units from 12 districts of Uttar Pradesh. For the purpose of this study, we presumed ‘January 2008’ as the starting point. It is to be noted that data for earlier SIAs were available in the CGPP India MIS. However, before January 2008, the CGPP’s MIS included more qualitative information rather than quantitative data. From October 2018, CGPP India withdrew CMCs from some areas and altered its intervention approach by introducing a low-intensity SM Net and intervening only through block-level functionaries. Therefore, we selected 'September 2017' as the endpoint of the study. In September 2017 (the endpoint of the study period), the CGPP had 1100 CMCs, deployed in 823 villages/urban wards from UP, reaching 522,000 households. Most of these CMC areas had a significantly high proportion (68%) of Muslims and a low female literacy level (45%) than non-intervention areas.

Similar to our previous study [16], we have not sampled and included all the 56 geographic areas (i.e., blocks/polio-planning units) where CGPP had its CLSM intervention during the study period (i.e., from January 2008 to September 2017). Similarly, we included all the 52 SIAs with a complete operation (booth-based and house-to-house vaccination) and covered all the geographic areas. Both the study areas (i.e., intervention and non-intervention areas) had the same number of polio SIAs (77), and an equal number of SIAs (52) are included in this study. Note also that 25 SIAs held during the study period (i.e., from January 2008 to September 2017) were excluded from the analysis because these SIAs had either partial operations (i.e., the SIAs included any one of the two main types of operations) or incomplete geographic coverage (i.e., the SIAs that did not cover all study units and limited to selected areas). Also, we excluded two CGPP blocks that were not covered at the start of the study period (Appendix Table 1).

Considering the date of 25th February 2012, when India became a polio-non-endemic county [19], we divided the SIAs of the entire study period into the following two periods: (1) polio-endemic period and (2) post-polio-endemic period. The 25 SIAs that took place before March 2012 are labeled as ‘Polio-endemic period SIAs’, whereas the ‘Post-polio-endemic period’ included 27 SIAs held from March 2012 to September 2017.

### Dependent variables

Using the CGPP MIS data, we computed various indicator variables to quantify the performance of polio SIAs (for both the fixed-site and house-to-house vaccination operations) and community engagement, separately for the intervention and non-intervention areas. We considered the following nine indicators as dependent variables for cross-temporal analysis:

(1) *Overall campaign coverage or SIA coverage* — This is the percentage of eligible children vaccinated (through polio booths and house-to-house activities) during an SIA. A total number of eligible children (i.e., number of children vaccinated in the previous SIA) is the denominator of this indicator.

(2) *Booth coverage* — The percentage of eligible children vaccinated at the polio SIA booths. Total number of children vaccinated in the preceding polio SIA is the denominator of this indicator.

(3) *Rate of ‘X’ houses generated at the beginning of an SIA* — The percentage of ‘X’ houses (i.e., the households with unvaccinated children or households with out-of-home/out-of village children or locked households) generated at the beginning of house-to-house vaccination of an SIA. The denominator of this indicator includes the total number of houses visited by house-to-house vaccination teams of an SIA. The numerator includes the number of “X” houses marked at the beginning phase (i.e., the first visit usually happens on Day 2 of an SIA) of house-to-house vaccination activity.

(4) *X-to-P conversion rate of an SIA* — The percentage of ‘X’ houses converted to ‘P’ (i.e., houses with all vaccinated children or absence of any eligible child for polio SIA vaccination) during a polio SIA. A total number of ‘X’ houses generated at the beginning phase of an SIA’s house-to-house activity is the denominator of this indicator.

(5) *Rate of remaining ‘X’ houses at the end of an SIA* — The percentage of remaining ‘X’ houses at the end of an SIA’s house-to-house activity. The denominator of this indicator includes the total number of houses visited by an SIA’s house-to-house vaccination teams.

(6) *Refusal rate at the start of house-to-house vaccination of an SIA* – This is the number of households who refused polio SIA vaccination at the beginning of an SIA’s house-to-house activity (Recorded as ‘XR houses’ in the tally sheets of vaccinators) against every 10,000 households visited by house-to-house vaccination teams.

(7) *Refusal-to-Acceptor conversion rate* —The percentage of resistant houses converted to acceptors during the house-to-house activities of an SIA. A total number of refusal houses generated at the beginning of the house-to-house vaccination activity denominates this indicator.

(8) *Refusal rate at the end of an SIA* – The number of households who refused polio vaccination at the end of an SIA’s house-to-house activity (Recorded as remaining ‘XR houses’ in the vaccinators’ tally sheets) against every 10,000 households visited by house-to-house vaccination teams.

(9) *Community Engagement Index (CEI) of polio SIA* — A composite indicator computed based on five selected indicators reflecting community engagement in polio SIAs (Refer to Choudhary et al., 2021 for computation details). The CEI reflects the overall level of community engagement in the polio SIAs and its values ranged from 0 to 1 (or 0 to 100%). CEI with zero value indicates no engagement of communities.

### Exploratory analysis and data cleaning

We carried out frequency analysis and box-plot analysis using MS Excel, SPSS and Tableau Desktop (public) Visualization software to identify the data with unexpected values (including typographical errors and outliers) for each indicator. We used the ‘Z score’ and box plots to check the outliers in the dataset (values less than − 2.68 or greater than 2.68). The data with extreme values were verified with the quarterly or annual narrative reports of the CGPP India, and unjustified outliers were replaced with the average values for all the study variables. Also, we performed a graphical analysis to observe trends and variations between the intervention and non-intervention areas.

### Generalized estimating equations (GEE) analysis

We used GEE-based analysis in STATA to assess the post-polio-endemic period difference in the nine indicators mentioned above of intervention and non-intervention areas. Similar to previous studies of Weiss et al. (2011) and Choudhary et al. [16], we performed GEE analysis to account for the longitudinal/panel nature of the data, including block/polio planning area level Intra-cluster correlation (ICC). We preferred ‘Quasi-likelihood under the independence model criterion (QIC)’ as the model selection method [15]. We considered the GEE model with the lowest QIC as the most appropriate one among the other competing models with different correlation structures (e.g., exchangeable, auto-regressive, unstructured etc.). In the GEE analysis, we assumed that the differences between the outcome indicators of intervention and non-intervention areas might vary by district, place of residence and time of year (quarter). We also assumed that there might be an interaction between the differences by intervention status (i.e., CLSM intervention/No intervention) and study district. That is, we expected the possibility that the effect of CMC activities in intervention areas, as compared to non-intervention areas, may get modified depending on the district being analyzed. The multivariate statistical analysis included the following independent variables: study district, place of residence of block/planning unit, time of year (and interaction terms if significant), and intervention status. The bivariate analysis compared indicators’ performance between intervention and non-intervention areas.

Further, we followed the recommendations of Bouttell et al. [20] and performed the different sensitivity analyses given below to assess the treatment effects of CLSM intervention on the SIA outcomes.

### Interrupted time-series analysis (ITSA)

ITSA analysis was performed to assess the extent of change (per SIA) and trends in the studied indicators. We used the ‘*itsa’* command in STATA and followed the guidelines of Linden [21]. The presence of autocorrelation in the data was checked through the ‘*actest’* command. If no autocorrelation was present for more than one leg, the default model with the ‘Newey’ option was selected. Otherwise, the *itsa* model included the ‘Prais’ option that adjusted the autocorrelation in the data. We performed 56 independent tests to assess the baseline comparability between the intervention area and each of the non-intervention areas. The final *itsa* model included the selected non-intervention areas with a *p*-value greater than 0.10 on both mean baseline difference (z) and mean baseline slope (z_t).

### Difference-in-differences (DID) analysis

Similar to our earlier analysis [16], we compared the differences between the polio-endemic and post-polio-endemic period outcomes. We used the ‘diff’ command in STATA, developed by Villa [22], and applied unadjusted, adjusted, and kernel PSM methods to estimate Difference-in-Differences (DID) treatment effects. Background characteristics that significantly differed between the intervention and non-intervention areas were considered covariates in the adjusted and Kernel PSM-based DID analysis. For the adjusted and kernel PSM based-analysis, we followed the recommendation of Oakes et al. [23] and included the covariates (independent variables) in the model, which predicts the exposure (to intervention) and not the outcome variable. The covariates were not identified through step-wise regression procedures or related techniques. Our possible covariates included selected characteristics of intervention and non-intervention area, i.e., average household size (Total individuals in a household), female literacy rate, percent Hindu/Muslim population and level of urbanization. A preliminary list of covariates to screen through further testing was identified through the t-test, using a 0.05 level of precision. We also performed balancing tests, using the ‘pstest’ command in STATA to check for covariate balance after the matching (for Kernel PSM-based analysis).

### Synthetic control method (SCM) based analysis

The SCM was applied to estimate the treatment effects based on an aggregated (weighted average) estimate of a combination of non-intervention areas that were similar to the intervention areas. Since the synth analysis in STATA allows only a single unit as an intervention [24], data from all CGPP intervention areas (i.e., CMC areas of all 56 blocks) were merged into a single unit and treated as ‘Intervention area’. In contrast, data from non-intervention areas of 56 blocks were treated as individual units of the Donor pool.^4^ We followed the analysis approaches and steps recommended in the literature related to the SCM [20, 25–27]. We used the *‘synth’* and *‘synth_runner’* packages in STATA to construct synthetic CMC areas and perform placebo tests for evaluating the significance of estimates. A synthetic intervention area was constructed based on the following three characteristics of both the intervention and non-intervention areas: percent urban population, female literacy rate, percent Hindu population. We used Root Mean Squared Predication error (RMSPE) of intervention areas to assess the goodness of fit and selected the SCM model with the lowest polio-endemic period RMSPE value. Ratios between the post-polio-endemic period RMSPE and the polio-endemic period RMSPE were used to determine the ill-fitting placebo runs. The non-intervention areas with the RMSPE ratio higher than the intervention areas were excluded from the final analysis.

Then we estimated the counterfactuals (i.e., the outcomes in the absence of CLSM intervention) through the following formula recommended for assessing a causal effect of an intervention or a program [28].

$$ \Delta =\left(\mathrm{Y}\ |\ \mathrm{P}=1\right)-\left(\mathrm{Y}\ |\ \mathrm{P}=0\right) $$

Where Δ denotes the causal effect of CLSM intervention (P) on an outcome (Y). (Y | *P* = 1) denotes an outcome with CLSM intervention and (Y | *P* = 0) to an outcome without the CLSM intervention (i.e., a counterfactual). Since the causal effect (Δ) and outcomes from intervention areas (Y | P = 1) were already assessed through other methods (defined earlier), we altered the positions of the formula elements in the following manner and assessed the counterfactuals.

$$ \left(\mathrm{Y}\ |\ \mathrm{P}=0\right)=\left(\mathrm{Y}\ |\ \mathrm{P}=1\right)-\Delta $$

Lastly, we attempted to estimate the population (i.e., number of households or under-five children) influenced by the CLSM intervention by multiplying the treatment effects with the actual population.

## Results

Targets and population reach of polio SIAs are presented in Table 1. The study areas (i.e., intervention and non-intervention areas) had a total population of 14 million in September 2017. Out of which, 3.8 million (or 27.1%) population was covered through the CLSM intervention. The total population of non-intervention areas has increased over time (from 7.1 million in 2011 to 10.4 million in 2017). In contrast, the intervention areas had an overall decrease of 1.6 million in the total population. In fact, the proportion of the total population (of a block) reached by CGPP’s CMCs has considerably declined from 43.5% in 2011 to 27.1% in 2017. This reduction (in the total population covered by CMCs) is the result of a gradual withdrawal of community-level SM Net intervention from the 250 CMC areas. Out of 1350 CMC areas of September 2011, the CGPP limited its CMC-level intervention to 1100 communities in September 2017.

**Table 1:** Population reach of polio SIAs conducted in the study areas

Information	Intervention areas	Non-intervention areas	Entire study area
Estimated total population:			
September 2011	5,468,832^a^	7,093,906^b^	12,562,738^c^
September 2014	4,789,282^a^	8,231,213^b^	13,020,475^c^
September 2017	3,846,832^a^	10,367,420^b^	14,214,252^c^
Average number of eligible under-five children:			
Polio-endemic period (Jan. 08 to Feb. 12)	635,922	1,805,079	2,441,001
Post-polio-endemic period (Mar. 12 to Sep. 17)	494,634	1,806,501	2,301,135
Entire study period (Jan. 08 to Sep. 17)	562,561	1,805,817	2,368,378
Average number of targeted households:			
Polio-endemic period (Jan. 08 to Feb. 12)	551,132	1,637,190	2,188,322
Post-polio-endemic period (Mar. 12 to Sep. 17)	541,853	1,889,686	2,431,538
Entire study period (Jan. 08 to Sep. 17)	546,314	1,768,294	2,314,607
Average number of polio vaccination booths:			
Polio-endemic period (Jan. 08 to Feb. 12)	1955	6045	8000
Post-polio-endemic period (Mar. 12 to Sep. 17)	1733	6960	8693
Entire study period (Jan. 08 to Sep. 17)	1839	6520	8360

a Estimated as: Total under-five population of the CMC areas (as per CGPP reports) divided by percent under-five population of CGPP districts (according to the Census of India, 2011) and multiplied by 100

b Computed as: Estimated total population of CMC areas subtracted from a total population of the study area

c Source: CGPP records (Total population of 56 CGPP blocks/polio planning units included in the study)

The polio eradication initiative in Uttar Pradesh, India, considers total vaccinated children of the previous campaign as a targeted population (i.e., eligible under-five children) for an SIA, and it may differ by SIAs. On average, 2.4 million under-five children from the entire study area (i.e., blocks covered by CGPP India in Uttar Pradesh India) were targeted for each polio SIA, out of which 5.6 lakhs (23.4%) under-five children were targeted in the intervention areas. As specified earlier, the decline in the number of targeted children from intervention areas is the result of a reduction in the number of CMC work areas. Ideally, the number of targeted children from non-CMC areas should have also increased over time, along with the increased total population. However, the Table displays a static number of under-five population for non-intervention areas, which might be the combined effect of varied coverage of the geographical areas (districts) by SIAs (See Appendix Table 1) and the definition of ‘eligible children’ that depends on the performance of previous SIAs.

About 1839 polio SIA booths were set up for vaccination in the intervention areas, and each booth covered about 306 eligible under-five children. At the same time, the non-intervention areas had 6520 polio vaccination booths that covered about 283 eligible children. About 2.3 million households were targeted (Visited by house-to-house vaccination teams) for polio vaccination in the entire study area. Of these, CMC areas covered 5.5 lakhs (23.6%) households. The intervention areas had a decline, but the non-intervention areas had an increase in the targeted households of SIAs in the post-polio-endemic period.

### Exploratory analysis

**Fig. 1:**
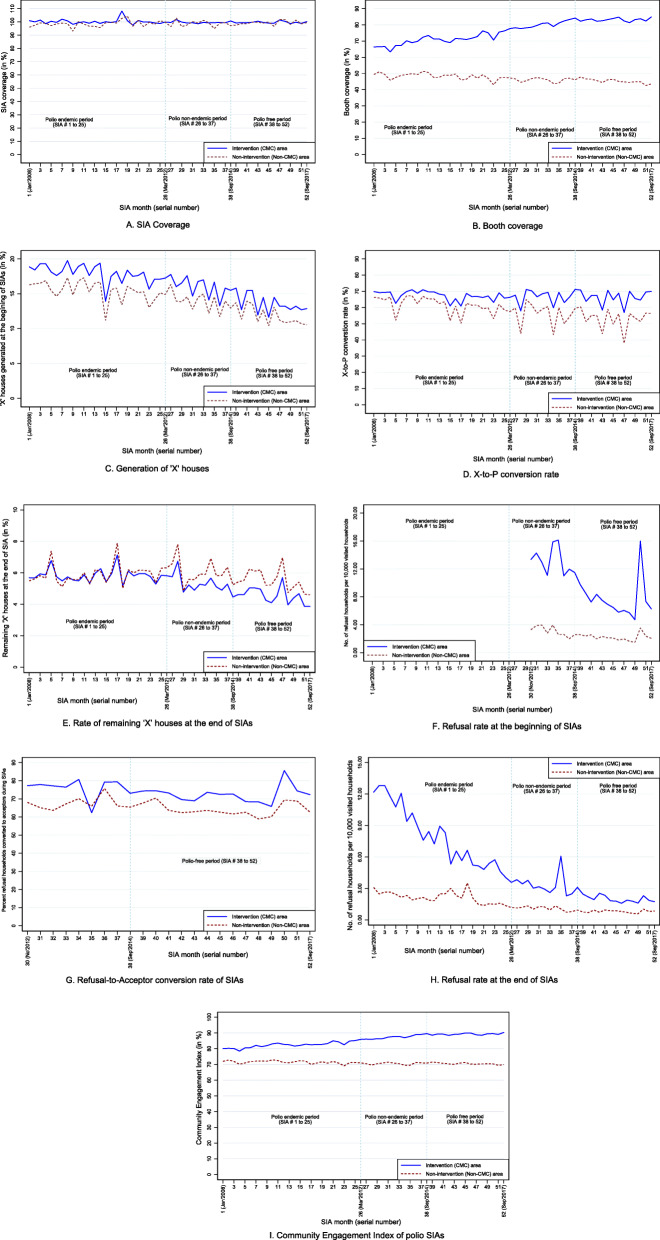
Trends in performance indicators and CEI of polio SIAs by Intervention Status, January 2008 to September 2017. Each line represents the mean value for each indicator. The mean value is calculated at the block level separately for CMC areas (Intervention areas) and non-CMC areas (Non-intervention areas). The blue line and broken brown line represents the intervention and non-intervention areas, respectively

The extent and trend of studied polio SIA indicators vary between the intervention and non-intervention areas (See Fig. 1 and Appendix Table 2). Outcomes of polio SIAs have improved over time for both the intervention and non-intervention areas. However, most of the indicators show better performance during the entire study period (i.e., January 2008 to September 2017) for intervention areas than non-intervention areas. The overall performance of polio SIAs (Measured as ‘percentage of estimated eligible children vaccinated in an SIA’) hovered around 100% during the entire study period. There was a very marginal difference of 1.3 percentage points between the two mean SIA coverage of intervention and non-intervention areas. Among all, the booth coverage indicator observed the highest gap of 29.4 percentage points between the two study arms with a divergent trend.

### Difference in post-polio-endemic period outcomes of polio SIAs

Table 2 summarizes the results of the GEE-based analysis that statistically tests the difference in post-polio-endemic period outcomes of intervention and non-intervention areas (Refer to Appendix Table 3a to 3i for indicator specific result). Except for the SIA-end refusal rate, the performance of all the studied indicators significantly varied (*p* < 0.05) between the intervention and non-intervention areas. Both the areas had a very high level of mean SIA coverage (> 99%), but there was a statistically significant difference of 0.7 percentage points between the intervention and non-intervention areas. Mean booth coverage of CLSM intervention areas was significantly higher (*p* < 0.01) by 36.4 percentage points than that of non-CMC areas. As the CLSM intervention areas had more polio high-risk areas, they had a significantly higher (*p* < 0.05) generation rate of ‘X’ houses and resistant houses (i.e., SIA-beginning refusal rate), compared to non-intervention areas. However, the intervention areas had a significantly higher (*p* < 0.05) conversion rate of ‘X-to-P’ and ‘Refusal-to-Acceptor’ that resulted in a significantly lower (*p* < 0.05) rate of remaining ‘X’ houses at the end of SIAs, compared to non-intervention areas. There was no significant difference (*p* > 0.05) between the SIA-end refusal rate of both areas. The intervention areas had a significantly (*p* < 0.01) higher level of community engagement (89.0%), by 18.2 percentage points, than non-intervention areas (70.8%).

**Table 2:** Mean values of outcome indicators and CEI of polio SIAs by intervention status, March 2012 to September 2017

Indicator	Mean (95% Confidence Interval)		
	Intervention Areas (n [blocks] = 56; Obs. per block = 27)	Non-intervention Areas (n [blocks] = 56; Obs. per block = 27)	*p*-value
SIA coverage (in %)	99.7 (99.6, 99.9)	99.0 (98.6, 99.3)	< 0.003^*^, ^**a^
Booth coverage (in %)	82.8 (82.5, 83.2)	46.4 (45.8, 46.9)	< 0.001^*^, ^**b^
Rate of ‘X’ houses generation at the beginning of house-to-house activities of SIAs (in %)	14.5 (14.3, 14.7)	12.9 (12.8, 13.1)	< 0.002^*^, ^**b^
Rate of X-to-P conversion during house-to-house activities of SIAs (in%)	66.3 (65.7, 66.9)	54.0 (53.2, 54.7)	< 0.001^*^, ^**a^
Rate of remaining ‘X’ houses at the end of house-to-house activities of SIAs (in %)	4.9 (4.8, 5.1)	5.9 (5.8, 6.0)	< 0.015^*^, ^**a^
Refusal rate (resistant households per 10,000 visited households) at the beginning of SIAs	8.0 (7.1, 8.8)	3.3 (3.0, 3.6)	< 0.003^*^, ^**a^
Refusal-to-Acceptor conversion rate of SIAs (in%)	73.7 (71.8, 75.5)	65.5 (63.6, 67.3)	< 0.004^*^, ^**a^
Refusal rate (resistant houses per 10,000 visited houses) at the end of SIAs	1.9 (1.5, 2.2)	1.2 (1.0, 1.3)	0.093^ns^
Community Engagement Index of polio SIAs (in %)	89.0 (88.8, 89.2)	70.8 (70.6, 71.1)	< 0.001^*^, ^**b^

* Bivariate analysis of an indicator by Intervention Status using generalized estimating equations (standard errors adjusted for clustering by block/Intervention Status)

**a Statistically significant difference (*p* < 0.05) in multivariate analysis of an Indicator by Intervention Status controlling for District, Place of residence and Time of year, using generalized estimating equations (standard errors adjusted for clustering by block/Intervention Status)

**b Statistically significant difference (*p* < 0.05) in multivariate analysis of an indicator Intervention Status controlling for District, Time of year and including interaction of District with Intervention Status, using generalized estimating equations (standard errors adjusted for clustering by block/Intervention Status)

^ns^ Statistically insignificant difference (*p* > 0.05) between intervention and non-intervention areas, in the bivariate analysis

### Effects of SM intervention on post-polio-endemic period outcomes of polio SIAs

Among the above discussed nine indicators, we excluded two indicators (Related to SIA-beginning vaccination status) and considered the following seven indicators for estimating the effects of CMSM intervention: (1) SIA coverage, (2) Booth coverage, (3) X-to-P conversion rate of an SIA, (4) Rate of remaining ‘X’ houses at the end of an SIA, (5) Refusal-to-Acceptor conversion rate of an SIA, (6) Refusal rate at the end of an SIA and (7) Community engagement index of polio SIAs.

Initial ITSA analysis, based on the selected non-intervention areas (with a baseline level of outcome similar to the intervention areas), found that the post-polio-endemic period trend of most of the above mentioned seven indicators differed from the baseline, i.e., polio-endemic period (See Appendix Table 4 and Appendix Fig. 1). The post-polio-endemic period trend of two indicators (i.e., Booth coverage and CEI) significantly (*p* < 0.05) varied between the intervention and non-intervention areas. Out of seven, four indicators (i.e., booth coverage; the rate of remaining ‘X’ house at the end of SIA, refusal rate at the SIA end and CEI) of intervention areas had improved performance over time; however, the trend did not vary by intervention status. The post-polio-endemic period booth coverage and CEI of intervention areas were significantly (*p* < 0.05) increased by 0.24 and 0.16 percentage points per SIA, respectively. Similarly, the intervention areas significantly reduced the rate of remaining ‘X’ and ‘XR’ (resistant) houses. Three indicators (i.e., SIA coverage, X-to-P conversion rate, and Refusal-to-Acceptor conversion rate) had insignificant change (*p* > 0.05) over time in both the intervention and non-intervention areas.

Table 3 provides mean values of polio SIA outcomes for seven indicators and the effects of community-level SM intervention on the post-polio-endemic period outcomes of the intervention, estimated based on different methods. The Table also presents the estimated counterfactual for each indicator. The intervention areas had greater positive outcomes than non-intervention areas. The difference between the outcomes of intervention and non-intervention areas was more significant during the post-polio-endemic period than the polio-endemic period (i.e., presumed pre-intervention period of the study). The estimated treatment effects of most of the outcomes vary by the estimation method. Table 4 presents the estimated population (i.e., number of households and under-five children) of intervention areas influenced/affected by the CLSM intervention during the post-polio-endemic period.

**Table 3:** **Mean values of polio SIA outcomes and estimated effects of community-level SM intervention on post-polio-endemic period outcomes**

Indicator	Mean values of SIA outcomes of:						Causal effect of community-level SM intervention on the post-polio-endemic period outcomes of intervention areas					Estimated mean counterfactual* (Range)
	Polio-endemic period (Jan. 2008 to Feb. 2012)			Post-polio-endemic period (Mar. 2012 to Sep. 2017)								
	Intervention areas	Non-intervention areas	Difference	Intervention areas	Non-intervention areas	Difference	Unadjusted DID	Adjusted DID	Kernel-PSM DID	SCM based effect	Mean effect (Range)	
1	2	3	4 = (2–3)	5	6	7 = (5–6)	8	9	10	11	12	13 = (5–12)
SIA coverage	100.3	98.5	1.8	99.7	99.0	0.7	−1.1^•^	−1.1^•^	−0.8 (NS)	0.09 (NS)	Not significant	Not calculated
Booth coverage	70.8	49.0	21.8	82.8	46.4	36.4	14.6	14.6	12.7	15.5	14.1 (12.7 to 15.5)	68.7 (67.3 to 70.1)
X-to-P conversion rate	66.7	61.0	5.7	66.3	54.0	12.3	6.6	6.6	7.3	5.2	6.3 (5.2 to 7.3)	60.1 (59.0 to 61.1)
Rate of remaining ‘X’ houses at the end of SIAs	5.95	6.02	−0.07	4.95	5.90	−0.95	−0.88	− 0.88	−1.12	− 0.88	−1.0 (− 0.88 to − 1.12)	5.95 (5.83 to 6.07)
Refusal-to-Acceptor conversion rate	76.02^a^	67.73 ^a^	8.29^a^	72.27^b^	64.20^b^	8.07^b^	−0.22^c^ (NS)	−0.22^c^ (NS)	7.40^c^	14.6^c^ (NS)	7.40^c^	64.87^c^
Refusal rate at the end of SIAs	7.307	2.602	4.705	2.040	1.241	0.799	−3.906	−3.906	−4.188	−7.678	−5.792 (−3.906 to −7.678)	7.832 (5.946 to 9.718)
Community Engagement Index (CEI) of polio SIAs	82.4	71.8	10.6	89.0	70.8	18.2	7.5	7.5	6.6	7.7	7.15 (6.6 to 7.7)	81.9 (81.3 to 82.4)

^•^ DID analysis explains very less proportion of (< 3%) total variance

a Observed outcome during the polio-non-endemic period (October 2012 to March 2014)

b Observed outcome during the polio-free period (April 2014 to September 2017)

c Estimated causal effect of SM Net intervention during the polio-free period (April 2014 to September 2017)

(NS) Statistically not significant (p-value > 0.05)

* Estimated counterfactual, i.e., “What would have happened to the outcome, if intervention areas had no community-level SM Net intervention”

**Table 4:** Estimated population of CMC areas influenced by the CLSM intervention

	Indicator	Mean (Range)
1	Number of eligible children from CMC areas that would not have been vaccinated at booths in each SIA of the post-polio-endemic period, in the absence of the SM intervention^*^	69,743 (62,819 to 76,668)
2	Number of additional houses from CMC areas that would have been remained ‘X’ at the end of each SIA of the post-polio-endemic period, in the absence of the SM intervention^†^	5419 (4768 to 6069)
3	Number of houses from CMC areas that would have been remained resistant at the end of each SIA of the post-polio-endemic period, in the absence of the SM intervention^‡^	424 (322 to 527)
4	Number of households from CMC areas that would not have been engaged in each SIA of the post-polio-endemic period, in the absence of the SM intervention^§^	38,742 (35,762 to 41,723)

* Estimated as: $$ \left\{\left(\begin{array}{c} Average\ number\ of\ eligible\ children\ of\\ {} post- polio- endemic\ period\ SIAs\ from\  CMC\  areas\end{array}\right)\ast \left(\begin{array}{c} Estimated\ treatment\ effect\  on\ \\ {} Booth\ coverage\end{array}\right)\right\}/100 $$

† Estimated as: $$ \left\{\left(\begin{array}{c} Average\ number\ of\ houses\ reached\ during\ SIAs\ of\\ {} post- polio- endemic\ period\ in\  CMC\  areas\end{array}\right)\ast \left(\begin{array}{c} Estimated\ treatment\ effect\  on\ \\ {} Rate\ of\ remaining\hbox{'}X\hbox{'} houses\  at\  the\  SIA\  end\end{array}\right)\right\}/100 $$

‡ Estimated as: $$ \left\{\left(\begin{array}{c} Average\ number\ of\ houses\ reached\ during\ SIAs\ of\\ {} post- polio- endemic\ period\ in\  CMC\  areas\end{array}\right)\ast \left(\begin{array}{c} Estimated\ counterfactual\ of\ \\ {} Refusl\ rate\  at\  the\  end\  of\  SIA\  end\end{array}\right)\right\}/\mathrm{10,000} $$

§ Estimated as: $$ \left\{\left(\begin{array}{c} Average\ number\ of\ targeted\ househods\ of\\ {} post- polio- endemic\ period\ SIAs\ from\  CMC\  areas\end{array}\right)\ast \left(\begin{array}{c} Estimated\ treatment\ effect\  on\ \\ {} Community\ Engagement\ Index\end{array}\right)\right\}/100 $$

#### SIA coverage

The Kernel-PSM DID and SCM-based analysis found no significant effect of CLSM intervention on the overall performance SIAs (SIA coverage) of intervention areas during the post-polio-endemic period. Whereas the unadjusted and adjusted DID methods found a significant negative effect of one percentage point. However, the test results are unreliable, as the analysis explained a tiny proportion (< 3%) of the total variance.

#### Booth coverage

Among the seven indicators, the CLSM intervention had the largest effect of 14.1 (Ranged from 12.7 to 15.5) percentage points on the post-polio-polio-endemic period booth coverage (i.e., percent eligible children vaccinated at polio SIA booths) of intervention areas. Thus, the achieved mean booth coverage of 82.8% in CMC areas would have gone down between the range of 67.3% to 70.1%. In numbers, in the absence of CLSM intervention during the post-polio-endemic period, about 69,743 (Range: 62,819 to 76,668) eligible under-five children from the CMC areas would not have been vaccinated at polio booths in each SIA.

#### X-to-P conversion and rate of remaining ‘X’ houses at the end of SIAs

In the absence of CLSM intervention in CMC areas, the percentage of ‘X’ houses converted to ‘P’ during the polio SIAs would have dropped in the range of 5.2 to 7.3 (Mean = 6.3) percentage points from the mean X-to-P conversion rate of 66.3% during the post-polio-endemic period. Consequently, the percentage of remaining ‘X’ houses at the SIA-end would have increased in the range of 0.88 to 1.12 percentage points from the observed mean value of 4.95. In other words, in addition to the 26,671 ‘X’ households (i.e., average ‘X’ households remained at the end of each SIAs), the absence of CLSM intervention would have added another 5419 (Range: 4768 to 6069) ‘X’ households to the remaining ‘X’ households.

#### Refusal-to-acceptor conversion rate and refusal rate at the end of SIAs

Since the CGPP MIS did not provide data for SIA beginning refusal rate for the polio-endemic period, the effects of CLSM intervention on Refusal-to-Acceptor conversion rate was assessed for the polio-free period (i.e., from April 2014 to September 2017). Without CLSM intervention, the percentage of refusal houses converted to polio acceptors would have significantly decreased by 7.4 percentage points from the observed mean conversion rate of 72.3%. Similarly, the observed mean ‘refusal rate at the end of polio SIAs’ of 2.040 would have also gone up in the range of 5.946 to 9.718 (Mean = 7.832) households among every 10,000 visited households. Thus, in absolute number, about 424 (Range: 322 to 527) households per 10,000 visited households would have remained resistant against the actual 143 remaining resistant households.

#### Community engagement index of polio SIAs

The CLSM intervention during the post-polio-endemic period had significantly increased the level of community engagement (measured through the Community Engagement Index) by 7.2 (Range: 6.6 to 7.7) percentage points in intervention areas. The achieved CEI of 89% in the intervention areas would have reduced to 81.3 (Range: 80.7 to 81.8) percentage points. In other words, the CLSM intervention increased engagement and participation by an estimated 38,724 (Range: 35,762 to 41,723) households from the CMC areas in each SIA of the post-polio-endemic period.

## Discussion

Study results show that the outcomes of polio SIAs in the CLSM intervention areas were equal to or exceeded the non-intervention areas’ outcomes, even though the intervention areas were the more challenging areas for vaccination. The absence of CLSM intervention during the post-polio-endemic period would have negatively impacted the performances of both the booth-based and house-to-house vaccination efforts of polio SIAs.

Dealing with resistance against polio vaccination was the unique salient point of the CLSM intervention. Reaching and converting about 424 (Range: 322 to 527) resistant households to polio accepters in each SIA from CMC areas (See Table 4) is a substantial achievement, as the study areas in the past experienced many instances where the snowball effect of one refusal family led to more refusals, and there were instances where entire communities were against the polio vaccination drive [6, 29, 30]. Eventually, the CLSM intervention increased engagement by 38,742 households, roughly translating into the inclusion of the same number of under-five children and a total population of 289,790 (Range: 267,500 to 312,088)^5^ in each of the polio SIAs of the post-polio-endemic period. The intervention reached and engaged the underserved and polio-high-risk populations.

The increased community engagements and polio SIA vaccination performances were achieved with some small economic investments. In addition to the operational cost incurred by national and state governments (i.e., Ministry of Health) and other polio partners, CGPP India’s CLSM intervention annually cost around USD 0.81^6^ (INR 52) per person reached. This rough estimation of intervention cost included non-vaccine operational costs (e.g., salaries/honorarium, travel, equipment/supplies, training, monitoring and evaluation, project activities, etc.). The number of persons reached included the intervention area population that comprised primary (under-fifteen year children and their parents/grandparents) and secondary/tertiary audiences of the CGPP. However, estimation of persons reached excluded CGPP functionaries’ efforts from non-CMC areas. Note that the CGPP India functionaries also assisted local health administration in planning and monitoring SIA operations in the non-intervention (i.e., non-CMC) areas of the CGPP blocks. Sometimes the block- and district-level functionaries of CGPP (i.e., BMCs and DMCs) even directly intervened in non-CMC areas to resolve the extreme situation of community-level resistance against polio vaccination [29].

### Limitations

This research is based on the assumption that the programmatic efforts of government and other polio partners (WHO/NPSP, and others) were equally implemented in both the intervention (CMC) and non-intervention (non-CMC) areas. As the study used the administrative data that might have the reporting bias of under-or over-reporting, it was assumed that the extent of data error or reporting bias was uniform for both the intervention and non-intervention areas. Like the previously conducted studies by Weiss et al. [15] and Choudhary et al. [16], this study also has a major limitation about the degree of comparability between the intervention and non-intervention areas. The study areas, particularly the CLSM intervention areas, were not randomly assigned and purposively included the polio-high-risk areas where a high proportion of communities were not accepting the polio vaccination. Thus, the actual effects of CLSM intervention might have been much more than estimated. The unavailability of quantitative data for the beginning period of CLSM intervention (i.e., from 2003 to 2008) only allowed us to estimate the counterfactual based on the assumed pre-intervention data of January 2008 to February 2012. Also, the unavailability of data on ‘Number of resistant (XR) houses generated at the beginning of SIAs’ for the polio-endemic period (i.e., before October 2012) restricted us to estimate the effects of CLSM intervention on ‘Refusal-to-Acceptor conversion rate’ only for the polio-free period (i.e., from April 2014 to September 2017) and not for the entire post-polio-endemic period.

Notwithstanding these limitations, the quasi-experimental design of the study that included the time-series data (with a non-equivalent comparison group) was able to determine whether a change takes place after the specified period or not, with greater internal validity. Furthermore, if a substantial change was observed during the subsequent observations after the baseline, then the design allowed us to reasonably conclude that the cause of the change was the intervention [31].

In this research, both the intervention and comparison areas were selected from the same district and both areas had similar programmatic interventions, except the additional CLSM activities in the intervention areas. The study used a single data source, and the SIAs were conducted simultaneously in both areas. Dissimilarities between the socio-economic profiles of intervention and non-intervention areas were the major confounding factors in our study. This research attempted to control for confounding and maximize internal validity by 1) Including the control areas that had no community-level intervention, 2) Including a substantial number of observations before the assumed treatment period, and 3) Statistically adjusting the effects of socio-economic characteristics of the study areas while assessing the treatment effects.

Our study findings are somewhat similar and supplement the previously conducted research that found that the SM Net initiative contributed to increasing the outcomes of polio SIAs [11, 15]. This research precisely attributes the outcomes of polio SIAs to the CLSM intervention. It provides evidence of an added value of having CLSM intervention even during the post-polio-endemic period. Further studies can precisely assess the health and economic benefits (e.g., number of polio cases averted, number of polio deaths averted, number of disability-adjusted life years averted, economic gain) of CLSM Net intervention, using the disease burden (polio incidence) data of longer duration from the intervention areas. Other research can test the replicability or adaptability of intensified CLSM intervention in other public health issues such as tuberculosis, breastfeeding and child feeding practices.

### Policy implications

Study findings indicate that the deployment of community-level paid volunteers, i.e., CMCs, was an effective strategy of SM Net initiative, even during the post-polio endemic period, to address access barriers and increase community engagement in polio SIAs. An intensified social mobilization or social and behavior change communication initiative with the deployment of dedicated community-level functionaries can be helpful to deal with the demand-side barriers in the utilization of public health services/schemes and achieve the desired outcomes of public health initiatives, especially in the areas with issues around acceptance of an intervention/program.

In the Indian scenario, a policy decision can be taken to deploy additional social mobilizers or volunteers under the national health system or through other systems, primarily to address specific public health challenges/issues, such as low routine immunization, harmful practices related to child care and feeding (including breastfeeding), Tuberculosis control, etc. Large-scale public health programs such as the National Health Mission (NHM) of India can apply the strategies and approaches of the SM Net initiative and build the communication skills as well as micro-planning capacities of Accredited Social Health Activists (ASHAs) to engage communities and perform their assigned tasks effectively. The ASHAs have a socio-economic profile similar to the CMCs of the SM Net initiative in Uttar Pradesh, India.

## Conclusions

This study found that the intervention areas had significantly better outcomes of polio SIAs than the non-intervention areas. The absence of CLSM intervention would have significantly decreased the level of community engagement and negatively impacted the performance of polio SIAs of the post-polio-endemic period. The intervention has significantly contributed to the polio eradication initiative of India by mobilizing the community and addressing resistance to polio vaccination. This study provides evidence of an added value of deploying additional human resources dedicated to social mobilization (e.g., CMCs of CGPP) to achieve the desired vaccination outcomes in hard-to-reach or programmatically challenging areas. Other public-health programs dealing with similar challenges can apply the learnings of the SM Net initiative of CGPP.

## Supplementary Information

**Additional file 1: Table S1.** List of polio SIAs along with their geographic coverage and inclusion status in the study, January 2008 to September 2017. **Table S2.** Aggregated performance of polio SIAs of the entire study period (January 2008 to September 2017) by intervention status. **Table S3a.** Mean SIA coverage by district, place of residence, time of year and intervention status, March 2012 to September 2017. **Table S3b.** Mean booth coverage by district, place of residence, time of year and intervention status, March 2012 to September 2017. **Table S3c.** Mean rate of ‘X’ houses generation at the beginning of house-to-house activities of SIAs by district, place of residence, time of year and intervention status, March 2012 to September 2017. **Table S3d.** Mean rate of X-to-P conversion during house-to-house activities (A and B team) of SIAs by district, place of residence, time of year and intervention status, March 2012 to September 2017. **Table S3e.** Mean rate of remaining ‘X’ houses at the end of house-to-house activities of SIAs by district, place of residence, time of year and intervention status, March 2012 to September 2017. **Table S3f.** Mean refusal rate (resistant households per 10,000 visited households) at the beginning of SIAs by district, place of residence, time of year and intervention status, October 2012 to September 2017. **Table S3g.** Mean Refusal-to-Acceptor conversion rate of SIAs by district, place of residence, time of year and intervention status, October 2012 to September 2017. **Table S3h.** Mean refusal rate (resistant houses per 10,000 visited houses) at the end of SIAs by district, place of residence, time of year and intervention status, October 2012 to September 2017. **Table S3i.** Mean level of community engagement in polio SIAs by district, place of residence, time of year and intervention status, March 2012 to September 2017. **Table S4.** Linear post-intervention (post-polio-endemic period) trend of polio SIA outcomes by intervention status.

**Additional file 2: Fig. S1.** Trends in actual and predicted values of seven indicators of intervention and selected non-intervention area, using two-group interrupted time-series analysis.

## Data Availability

As the study used the administrative data of CGPP India, the datasets analyzed during the current study are not publicly available. Public access to the study database is closed. However, data are available from the corresponding author on reasonable request.

## Abbreviations

CEI: Community Engagement Index
CGPP: CORE Group Polio Project
CLSM: Community-level Social Mobilization
CMC: Community Mobilization Coordinators
DID: Difference-in-Differences
GEE: Generalized Estimating Equations
ITSA: Interrupted Time-Series Analysis
PSM: Propensity-Score Matching
SBCC: Social and behavior change communication
SCM: Synthetic Control Method
SIA: Supplementary Immunization Activity
SM: Social Mobilization
SM Net: Social Mobilization Network
UP: Uttar Pradesh

## Footnotes

1 Polio planning units – are the smaller cluster of urban areas (urban wards) from administrative block or a city [9].

2 HRAs were designated on the basis of multiple indicators not just refusals and hard to reach areas

3 In polio SIAs, fixed site or booth-based vaccination refers to a process of dispensing oral polio vaccine to eligible children at kiosks (booths) set up at fixed sites in a community. These booths are temporary and located at different places such as health facilities, educational institutions, residential premises and transit places like railway stations, bus stops.

4 Donor pool – refers to a set of potential control areas [25] . In this study, donor pool included the villages/urban wards without community-level SM Net intervention (also known as non-CMC areas) from a block/polio-planning unit.

5 *Estimated population of CMC areas that would not have been engaged in the absence of CLSM intervention* ≈ *Estimated number of households that would not have been engaged from CMC areas* ∗ *Average household size of CMC areas*

6 $$ \mathrm{Estimated}\ \mathrm{cost}\ \mathrm{per}\ \mathrm{per}\mathrm{son}\ \mathrm{reached}\ \left(\mathrm{Annual}\right)=\frac{Annual\ budget\ of\ the\ intervention}{Total\ population\ reached\  by\  the\ intervention} $$
