# Supplementary material for: Effectiveness of a community-level social mobilization intervention in achieving the outcomes of polio vaccination campaigns during the post-polio-endemic period: Evidence from CORE Group polio project in Uttar Pradesh, India
Source: BMC Public Health. 2021 Jul 10;21:1371. doi: 10.1186/s12889-021-11425-0 (PMC8272292; doi:10.1186/s12889-021-11425-0)
Supplement: Supplementary file 1 — Additional file 1: Table S1. List of polio SIAs along with their geographic coverage and inclusion status in the study, January 2008 to September 2017. Table S2. Aggregated performance of polio SIAs of the entire study period (January 2008 to September 2017) by intervention status. Table S3a. Mean SIA coverage by district, place of residence, time of year and intervention status, March 2012 to September 2017. Table S3b. Mean booth coverage by district, place of residence, time of year and intervention status, March 2012 to September 2017. Table S3c. Mean rate of ‘X’ houses generation at the beginning of house-to-house activities of SIAs by district, place of residence, time of year and intervention status, March 2012 to September 2017. Table S3d. Mean rate of X-to-P conversion during house-to-house activities (A and B team) of SIAs by district, place of residence, time of year and intervention status, March 2012 to September 2017. Table S3e. Mean rate of remaining ‘X’ houses at the end of house-to-house activities of SIAs by district, place of residence, time of year and intervention status, March 2012 to September 2017. Table S3f. Mean refusal rate (resistant households per 10,000 visited households) at the beginning of SIAs by district, place of residence, time of year and intervention status, October 2012 to September 2017. Table S3g. Mean Refusal-to-Acceptor conversion rate of SIAs by district, place of residence, time of year and intervention status, October 2012 to September 2017. Table S3h. Mean refusal rate (resistant houses per 10,000 visited houses) at the end of SIAs by district, place of residence, time of year and intervention status, October 2012 to September 2017. Table S3i. Mean level of community engagement in polio SIAs by district, place of residence, time of year and intervention status, March 2012 to September 2017. Table S4. Linear post-intervention (post-polio-endemic period) trend of polio SIA outcomes by intervention status. [file 12889_2021_11425_MOESM1_ESM.docx]

**Appendix Tables**

**Appendix Table 1. List of polio SIAs along with their geographic coverage and inclusion status in the study, January 2008 to September 2017**

| **No.** | **Polio SIA dates** | **No. of CGPP districts covered** | **No. of CGPP blocks/ polio planning units** | **SIA type** | **Inclusion/ Exclusion status** | **SIA No.** |
| --- | --- | --- | --- | --- | --- | --- |
| **Study period 1 - Polio-endemic period** (January 2008 to February 2012) | | | | | | |
| 1 | 06 January 2008 | 12 | 56 | NID | Included | 1 |
| 2 | 10 February 2008 | 12 | 56 | NID | Included | 2 |
| 3 | 30 March 2008 | 12 | 56 | SNID | Included | 3 |
| 4 | 27 April 2008 | 12 | 56 | SNID | Included | 4 |
| 5 | 01 June 2008 | 12 | 56 | SNID | Included | 5 |
| 6 | 29 June 2008 | 12 | 56 | SNID | Included | 6 |
| 7 | 27 July 2008 | 05 | 24 | SNID | Excluded |  |
| 8 | 03 August 2008 | 04 | 20 | SNID | Excluded |  |
| 9 | 26 August 2008 | 05 | 24 | SNID | Excluded |  |
| 10 | 14 September 2008 | 12 | 56 | SNID | Included | 7 |
| 11 | 18 October 2008 | 08 | 40 | SNID | Excluded |  |
| 12 | 16 November 2008 | 12 | 56 | SNID | Included | 8 |
| 13 | 21 December 2008 | 12 | 56 | NID | Included | 9 |
| 14 | 01 February 2009 | 12 | 56 | NID | Included | 10 |
| 15 | 01 March 2009 | 12 | 56 | SNID | Included | 11 |
| 16 | 05 April 2009 | 09 | 36 | Mop Up | Excluded |  |
| 17 | 21 April 2009 | 01 | 06 | SNID | Excluded |  |
| 18 | 17 May 2009 | 09 | 46 | SNID | Excluded |  |
| 19 | 05 July 2009 | 12 | 56 | SNID | Included | 12 |
| 20 | 26 July 2009 | 04 | 19 | SNID | Excluded |  |
| 21 | 09 August 2009 | 12 | 56 | SNID | Included | 13 |
| 22 | 10 September 2009 | 12 | 56 | SNID | Excluded  (No booth day activity) |  |
| 23 | 04 October 2009 | 10 | 50 | Mop Up | Excluded |  |
| 24 | 08 November 2009 | 12 | 56 | SNID | Included | 14 |
| 25 | 06 December 2009 | 10 | 50 | SNID | Excluded |  |
| 26 | 10 January 2010 | 12 | 56 | NID | Included | 15 |
| 27 | 07 February 2010 | 12 | 56 | NID | Included | 16 |
| 28 | 21 March 2010 | 10 | 50 | SNID | Excluded |  |
| 29 | 25 April 2010 | 10 | 50 | SNID | Excluded |  |
| 30 | 23 May 2010 | 12 | 56 | SNID | Included | 17 |
| 31 | 20 June 2010 | 09 | 46 | SNID | Excluded |  |
| 32 | 18 July 2010 | 01 | 04 | SNID | Excluded |  |
| 33 | 19 September 2010 | 12 | 58 | SNID | Included | 18 |
| 34 | 14 November 2010 | 12 | 58 | SNID | Included | 19 |
| 35 | 23 January 2011 | 12 | 58 | NID | Included | 20 |
| 36 | 27 February 2011 | 12 | 58 | NID | Included | 21 |
| 37 | 27 March 2011 | 12 | 58 | SNID | Included | 22 |
| 38 | 24 April 2011 | 10 | 50 | SNID | Excluded |  |
| 39 | 22 May 2011 | 10 | 50 | SNID | Excluded |  |
| 40 | 26 June 2011 | 12 | 58 | SNID | Included | 23 |
| 41 | 26 July 2011 | 01 | 10 | Mop Up | Excluded |  |
| 42 | 21 August 2011 | 10 | 50 | SNID | Excluded |  |
| 43 | 25 September 2011 | 12 | 58 | SNID | Included | 24 |
| 44 | 13 November 2011 | 08 | 40 | SNID | Excluded |  |
| 45 | 15 January 2012 | 10 | 50 | SNID | Excluded |  |
| 46 | 5 & 19 February 2012 | 12 | 58 | NID | Included | 25 |
| **Study period 2 – Post-polio-endemic period** (March 2012 to September 2017) | | | | | | |
| 47 | 18 March 2012 | 12 | 58 | SNID | Included | 26 |
| 48 | 15 April 2012 | 12 | 58 | NID | Included | 27 |
| 49 | 17 June 2012 | 12 | 58 | SNID | Included | 28 |
| 50 | 09 September 2012 | 12 | 58 | SNID | Included | 29 |
| 51 | 04 November 2012 | 12 | 58 | SNID | Included | 30 |
| 52 | 20 January 2013 | 12 | 58 | NID | Included | 31 |
| 53 | 24 February 2013 | 12 | 58 | NID | Included | 32 |
| 54 | 07 April 2013 | 12 | 58 | SNID | Included | 33 |
| 55 | 16 June 2013 | 12 | 58 | SNID | Included | 34 |
| 56 | 15 September 2013 | 12 | 58 | SNID | Excluded  (No booth day activity) |  |
| 57 | 24 November 2013 | 12 | 58 | SNID | Included | 35 |
| 58 | 19 January 2014 | 12 | 58 | NID | Included | 36 |
| 59 | 23 February 2014 | 12 | 58 | NID | Included | 37 |
| 60 | 24 April 2014 | 10 | 48 | SNID | Excluded |  |
| 61 | 14 September 2014 | 12 | 58 | SNID | Included | 38 |
| 62 | 16 November 2014 | 12 | 58 | SNID | Included | 39 |
| 63 | 18 January 2015 | 12 | 58 | NID | Included | 40 |
| 64 | 22 February 2015 | 12 | 58 | NID | Included | 41 |
| 65 | 26 April 2015 | 12 | 58 | SNID | Included | 42 |
| 66 | 21 June 2015 &  13 September 2015 | 12 | 58 | SNID | Included | 43 |
| 67 | 22 November 2015 | 12 | 58 | SNID | Included | 44 |
| 68 | 13 December 2015 | 04 | 20 | SNID | Excluded |  |
| 69 | 17 January 2016 | 12 | 58 | NID | Included | 45 |
| 70 | 21 February 2016 | 12 | 58 | NID | Included | 46 |
| 71 | 16 April 2016 | 11 | 52 | SNID | Excluded  (No booth day activity) |  |
| 72 | 29 May 2016 | 12 | 58 | SNID | Included | 47 |
| 73 | 25 September 2016 | 12 | 58 | SNID | Included | 48 |
| 74 | 29 January 2017 | 12 | 58 | NID | Included | 49 |
| 75 | 02 April 2017 | 12 | 58 | NID | Included | 50 |
| 76 | 02 July 2017 | 12 | 58 | SNID | Included | 51 |
| 77 | 17 September 2017 | 12 | 58 | SNID | Included | 52 |
| NID = National Immunization Day; SNID = Sub-National Immunization Day | | | | | | |

**Appendix Table 2. Aggregated performance of polio SIAs of the entire study period (January 2008 to September 2017) by intervention status**

| **SIA performance Indicator** | **Entire study area (n=5824)** | | **Intervention areas**  **(n = 2912)** | | **Non-intervention areas (n = 2912)** | | **Difference between intervention and non-intervention areas** | | | | |
| --- | --- | --- | --- | --- | --- | --- | --- | --- | --- | --- | --- |
|  | **Mean** | **SE** | **Mean** | **SE** | **Mean** | **SE** | **Mean** | **SE** | **95% CI** | **t stat.** | **p-value** |
| SIA coverage ( in %) | 99.4 | 0.076 | 100.0 | 0.0.072 | 98.7 | 0.221 | 1.3 | 0.151 | 0.97, 1.56 | 8.410 | <0.001 |
| Booth coverage ( in %) | 62.3 | 0.239 | 77.1 | 0.177 | 47.6 | 0.221 | 29.4 | 0.283 | 28.9, 30.0 | 103.867 | <0.001 |
| Generation rate of ‘X’ houses (in %) | 15.1 | 0.050 | 16.1 | 0.075 | 14.2 | 0.062 | 1.9 | 0.097 | 1.7, 2.1 | 19.557 | <0.001 |
| X-to-P conversion rate of an SIA (in %) | 61.9 | 0.177 | 66.5 | 0.204 | 57.3 | 0.262 | 9.2 | 0.332 | 8.5, 9.8 | 27.640 | <0.001 |
| Rate of remaining ‘X’ houses at the end of an SIA (in %) | 5.69 | 0.030 | 5.43 | 0.044 | 5.95 | 0.041 | -0.52 | 0.060 | -0.64, -0.41 | -8.705 | <0.001 |
| Refusal rate at the beginning of house-to-house activity of an SIA (number of resistant households per 10,000 visited households)  *(From Oct. 2012 to Sep. 2017)* | 5.64 | 0.234 | 7.97 | 0.428 | 3.31 | 0.168 | 4.66 | 0.460 | 3.76, 5.57 | 10.146 | <0.001 |
|  | (n=2576) | | (n=1288) | | (n=1288) | |  |  |  |  |  |
| Refusal-to-Acceptor conversion rate of an SIA (in %)  *(From Oct. 2012 to Sep. 2017)* | 69.5 | 0.670 | 73.7 | 0.945 | 65.5 | 0.933 | 8.2 | 1.328 | 5.6, 10.8 | 6.175 | <0.001 |
|  | (n=2171) | | (n=1065) | | (n=1106) | |  |  |  |  |  |
| Refusal rate at the end of an SIA (number of resistant households per 10,000 visited households)  *(From Oct. 2012 to Sep. 2017)* | 1.54 | 0.096 | 1.89 | 0.176 | 1.18 | 0.075 | 0.71 | 0.191 | 0.34, 1.09 | 3.742 | 0.001 |
|  | (n=2576) | | (n=1288) | | (n=1288) | |  |  |  |  |  |
| Community Engagement Index | 78.6 | 0.120 | 85.2 | 0.093 | 71.3 | 0.111 | 14.5 | 0.145 | 14.25, 14.8 | 100.323 | <0.001 |
|  | (n=5824) | | (n=2912) | | (n=2912) | |  |  |  |  |  |
| t statistic based on Independent samples t-test for equality of means | | | | | | | | | | | |

**Appendix Table 3a. Mean SIA coverage by district, place of residence, time of year and intervention status, March 2012 to September 2017**

|  | **By:** | **SIA Coverage (in %)**  **(95% Confidence Interval)** | |  |  |
| --- | --- | --- | --- | --- | --- |
|  |  | **Intervention Areas**  (n [blocks]= 56;  Obs. per block=27**)** | **Non-intervention Areas**  (n [blocks]= 56;  Obs. per block=27**)** | **p-value** |  |
|  | **Overall** | 99.7 (99.6, 99.9) | 99.0 (98.6, 99.3) | <0.003^*^, ^**^ |  |
|  |  |  |  |  |  |
|  | **District** |  |  |  |  |
|  | Baghpat | 99.7 (99.2, 100.1) | 99.1 (98.1, 100.1) | 0.204^ns^ |  |
|  | Bareilly | 99.5 (99.0, 99.9) | 99.7 (98.7, 100.6) | 0.468^ns^ |  |
|  | Mau | 100.2 (99.8, 100.7) | 100.2 (99.7, 100.7) | 0.943^ns^ |  |
|  | Meerut | 100.1 (99.6, 100.7) | 99.0 (97.9, 100.1) | 0.006^**^ |  |
|  | Moradabad | 99.4 (98.9, 99.8) | 95.3 (93.2, 97.4) | 0.001^**^ |  |
|  | Muzaffarnagar | 100.0 (99.4, 100.5) | 99.7 (98.8, 100.7) | 0.477^ns^ |  |
|  | Rampur | 99.7 (99.2, 100.2) | 99.1 (98.3, 99.8) | <0.001^**^ |  |
|  | Saharanpur | 100.0 (99.0, 101.0) | 98.8 (97.3, 100.4) | 0.023^**^ |  |
|  | Sambhal | 99.4 (98.8, 99.9) | 97.9 (96.3, 99.5) | 0.015^**^ |  |
|  | Shahjahanpur | 99.7 (99.3, 100.0) | 100.1 (99.3, 100.9) | 0.122^ns^ |  |
|  | Shamli | 99.6 (98.9, 100.3) | 100.0 (98.5, 101.5) | 0.369^ns^ |  |
|  | Sitapur | 99.7 (99.2, 100.1) | 99.6 (98.9, 100.3) | 0.867^ns^ |  |
|  |  |  |  |  |  |
|  | **Place of residence** |  |  |  |  |
|  | Rural | 99.8 (99.6, 99.9) | 99.3 (99.0, 99.6) | 0.002^**^ |  |
|  | Urban | 99.5 (98.9, 100.1) | 96.2 (94.1, 98.2) | 0.002^**^ |  |
|  |  |  |  |  |  |
|  | **Time of year** |  |  |  |  |
|  | January to March | 99.4 (99.2, 99.6) | 98.8 (98.3, 99.3) | 0.093^ns^ |  |
|  | April to June | 100.1 (99.8, 100.3) | 100.0 (99.3, 100.7) | 0.925^ns^ |  |
|  | July to September | 100.1 (99.7, 100.5) | 98.8 (98.1, 99.5) | <0.001^**^ |  |
|  | October to December | 99.4 (98.9, 99.9) | 97.6 (96.5, 98.6) | <0.001^**^ |  |
|  | * Bivariate analysis of SIA Coverage by Intervention Status using generalized estimating equations (standard errors adjusted for clustering by block/Intervention Status)  ** Statistically significant difference (p<0.05) in multivariate analysis of SIA Coverage by Intervention Status controlling for District, Place of residence and Time of year, using generalized estimating equations (standard errors adjusted for clustering by block/Intervention Status).  ^ns^ Statistically insignificant difference (p>0.05) between intervention and non-intervention areas, in the multivariate analysis | | | |  |

**Appendix Table 3b. Mean booth coverage by district, place of residence, time of year and intervention status, March 2012 to September 2017**

|  | **By:** | **Booth Coverage, (in %)**  **(95% Confidence Interval)** | |  |  |
| --- | --- | --- | --- | --- | --- |
|  |  | **Intervention Areas**  (n [blocks]= 56;  Obs. per block=27**)** | **Non-intervention Areas**  (n [blocks]= 56;  Obs. per block=27**)** | **p-value** |  |
|  | **Overall** | 82.8 (82.5, 83.2) | 46.4 (45.8, 46.9) | < 0.001^*^,^**^ |  |
|  |  |  |  |  |  |
|  | **District** |  |  |  |  |
|  | Baghpat | 83.4 (82.9, 84.0) | 46.6 (45.9, 47.4) | < 0.001^**^ |  |
|  | Bareilly | 84.3 (83.3, 85.3) | 48.2 (47.3, 49.1) | < 0.001^**^ |  |
|  | Mau | 89.0 (87.6, 90.3) | 40.4 (39.8, 41.1) | < 0.001^**^ |  |
|  | Meerut | 82.1 (81.2, 83.0) | 59.2 (57.6, 60.8) | < 0.001^**^ |  |
|  | Moradabad | 77.8 (76.9, 78.8) | 51.4 (50.0, 52.8) | < 0.001^**^ |  |
|  | Muzaffarnagar | 83.5 (82.2, 84.8) | 43.5 (42.7, 44.4) | < 0.001^**^ |  |
|  | Rampur | 84.1 (83.4, 84.7) | 45.5 (44.5, 46.4) | < 0.001^**^ |  |
|  | Saharanpur | 85.6 (84.2, 86.9) | 55.2 (53.8, 56.5) | < 0.001^**^ |  |
|  | Sambhal | 76.1 (75.3, 76.8) | 55.3 (53.9, 56.8) | < 0.001^**^ |  |
|  | Shahjahanpur | 85.7 (84.8, 86.6) | 42.8 (41.6, 44.1) | < 0.001^**^ |  |
|  | Shamli | 82.3 (80.9, 83.6) | 46.3 (44.8, 47.8) | < 0.001^**^ |  |
|  | Sitapur | 82.0 (81.3, 82.8) | 26.4 (25.5, 27.3) | < 0.001^**^ |  |
|  |  |  |  |  |  |
|  | **Place of residence** |  |  |  |  |
|  | Rural | 83.5 (83.1, 83.8) | 45.9 (45.3, 46.5) | < 0.001^* a^ |  |
|  | Urban | 77.8 (76.8, 78.9) | 50.2 (48.7, 51.7) | < 0.001^* a^ |  |
|  |  |  |  |  |  |
|  | **Time of year** |  |  |  |  |
|  | January to March | 83.5 (83.0, 84.0) | 47.3 (46.4, 48.2) | < 0.001^**^ |  |
|  | April to June | 81.9 (81.3, 82.5) | 45.7 (44.7, 46.7) | < 0.001^**^ |  |
|  | July to September | 83.4 (82.7, 84.2) | 45.1 (43.9, 46.3) | < 0.001^**^ |  |
|  | October to December | 82.4 (81.6, 83.2) | 47.1 (45.6, 48.6) | < 0.001^**^ |  |
|  | * Bivariate analysis of Booth Coverage by Intervention Status using generalized estimating equations (standard errors adjusted for clustering by block/Intervention Status)  ** Statistically significant difference (p<0.01) in multivariate analysis of Booth Coverage by Intervention Status controlling for District, Time of year and including interaction of District with Intervention Status, using generalized estimating equations (standard errors adjusted for clustering by block/Intervention Status).  a Not included in the multivariate GEE analyses, as the initial analysis (i.e., the model included: Booth coverage, Intervention status and Place of residence) found an insignificant difference (p=0.684) between the Booth coverage of rural and urban areas. | | | |  |

**Appendix Table 3c. Mean rate of ‘X’ houses generation at the beginning of house-to-house activities of SIAs by district, place of residence, time of year and intervention status, March 2012 to September 2017**

|  | **By:** | **Percentage of ‘X’ houses generated at the beginning phase of house-to-house activities of SIAs**  **(95% Confidence Interval)** | |  |  |
| --- | --- | --- | --- | --- | --- |
|  |  | **Intervention Areas**  (n [blocks]= 56;  Obs. per block=27**)** | **Non-intervention Areas**  (n [blocks]= 56;  Obs. per block=27**)** | **p-value** |  |
|  | **Overall** | 14.5 (14.3, 14.7) | 12.9 (12.8, 13.1) | <0.002^*^, ^**^ |  |
|  |  |  |  |  |  |
|  | **District** |  |  |  |  |
|  | Baghpat | 19.3 (18.8, 19.8) | 15.1 (14.7, 15.5) | <0.001^**^ |  |
|  | Bareilly | 13.0 (12.5, 13.5) | 11.6 (11.2, 12.0) | 0.355^ns^ |  |
|  | Mau | 10.2 (09.7, 10.6) | 09.4 (09.0, 09.7) | 0.290^ns^ |  |
|  | Meerut | 15.1 (14.6, 15.6) | 12.1 (11.7, 12.5) | 0.003^**^ |  |
|  | Moradabad | 14.7 (14.3, 15.0) | 13.0 (12.5, 13.5) | 0.002^**^ |  |
|  | Muzaffarnagar | 17.2 (16.6, 17.7) | 15.0 (14.5, 15.5) | 0.122^ns^ |  |
|  | Rampur | 10.7 (10.3, 11.2) | 10.2 (09.9, 10.6) | 0.444^ns^ |  |
|  | Saharanpur | 14.3 (13.8, 14.7) | 13.5 (13.1, 14.0) | 0.222^ns^ |  |
|  | Sambhal | 15.8 (15.4, 16.2) | 14.7 (14.3, 15.2) | 0.144^ns^ |  |
|  | Shahjahanpur | 13.4 (12.9, 13.9) | 12.6 (12.1, 13.1) | 0.511^ns^ |  |
|  | Shamli | 17.2 (16.5, 17.9) | 16.2 (15.3, 17.0) | 0.028^**^ |  |
|  | Sitapur | 12.4 (12.0, 12.8) | 12.2 (11.9, 12.5) | 0.770^ns^ |  |
|  |  |  |  |  |  |
|  | **Place of residence** |  |  |  |  |
|  | Rural | 14.5 (14.2, 14.7) | 12.8 (12.7, 13.0) | 0.003^* a^ |  |
|  | Urban | 14.7 (14.4, 15.1) | 13.6 (13.1, 14.1) | 0.061^* a^ |  |
|  |  |  |  |  |  |
|  | **Time of year** |  |  |  |  |
|  | January to March | 14.2 (13.9, 14.5) | 12.9 (12.6, 13.1) | <0.001^**^ |  |
|  | April to June | 14.6 (14.2, 15.0) | 13.1 (12.8, 13.4) | <0.001^**^ |  |
|  | July to September | 13.8 (13.4, 14.2) | 11.8 (11.5, 12.1) | <0.001^**^ |  |
|  | October to December | 15.8 (15.3, 16.2) | 14.0 (13.7, 14.3) | <0.001^**^ |  |
|  | * Bivariate analysis of ‘X’ houses generation rate by Intervention Status using generalized estimating equations (standard errors adjusted for clustering by block/Intervention Status).  ** Statistically significant difference (p<0.05) in multivariate analysis of ‘X’ houses generation rate by Intervention Status controlling for District and Time of year and including interaction of District with Intervention Status, using generalized estimating equations (standard errors adjusted for clustering by block/Intervention Status).  ^ns^ Statistically insignificant difference (p>0.05) between intervention and non-intervention areas, in the multivariate analysis  a Not included in the multivariate GEE analyses, as the initial analysis (i.e., the model included: ‘X’ houses generation rate, Intervention status and Place of residence) found insignificant difference (p=0.282) between the ‘X’ houses generation rate of rural and urban areas. | | | |  |

**Appendix Table 3d. Mean rate of X-to-P conversion during house-to-house activities (A and B team) of SIAs by district, place of residence, time of year and intervention status, March 2012 to September 2017**

|  | **By:** | **Percentage of ‘X’ houses converted to P during SIAs**  **(95% Confidence Interval)** | |  |  |
| --- | --- | --- | --- | --- | --- |
|  |  | **Intervention Areas**  (n [blocks]= 56;  Obs. per block=27**)** | **Non-intervention Areas**  (n [blocks]= 56;  Obs. per block=27**)** | **p-value** |  |
|  | **Overall** | 66.3 (65.7, 66.9) | 54.0 (53.2, 54.7) | <0.001^*^,^**^ |  |
|  |  |  |  |  |  |
|  | **District** |  |  |  |  |
|  | Baghpat | 65.5 (64.1, 67.0) | 56.2 (54.0, 58.3) | 0.011^**^ |  |
|  | Bareilly | 60.3 (58.2, 62.3) | 46.6 (45.1, 48.2) | 0.014^**^ |  |
|  | Mau | 72.5 (70.1, 74.9) | 37.8 (35.5, 40.2) | <0.001^**^ |  |
|  | Meerut | 68.7 (67.1, 70.4) | 60.7 (58.4, 63.0) | 0.007^**^ |  |
|  | Moradabad | 75.9 (74.0, 77.8) | 69.8 (67.4, 72.2) | 0.004^**^ |  |
|  | Muzaffarnagar | 56.9 (55.1, 58.6) | 47.1 (45.1, 49.1) | 0.053^ns^ |  |
|  | Rampur | 73.4 (72.1, 74.7) | 59.3 (57.6, 60.9) | <0.001^**^ |  |
|  | Saharanpur | 77.2 (75.7, 78.7) | 70.8 (68.6, 73.0) | <0.001^**^ |  |
|  | Sambhal | 64.4 (63.0, 65.8) | 51.3 (49.5, 53.0) | 0.001^**^ |  |
|  | Shahjahanpur | 53.2 (51.3, 55.1) | 42.1 (40.1, 44.1) | 0.058^ns^ |  |
|  | Shamli | 62.7 (60.4, 65.0) | 54.6 (51.5, 57.6) | 0.004^**^ |  |
|  | Sitapur | 67.6 (66.6, 68.5) | 52.9 (51.6, 54.2) | <0.001^**^ |  |
|  |  |  |  |  |  |
|  | **Place of residence** |  |  |  |  |
|  | Rural | 64.6 (64.0, 65.2) | 51.7 (51.0, 52.4) | <0.001^**^ |  |
|  | Urban | 80.2 (79.1, 81.4) | 73.1 (71.2, 75.0) | <0.001^**^ |  |
|  |  |  |  |  |  |
|  | **Time of year** |  |  |  |  |
|  | January to March | 66.1 (65.1, 67.2) | 53.9 (52.7, 55.1) | <0.001^**^ |  |
|  | April to June | 62.7 (61.6, 63.9) | 49.3 (47.8, 50.7) | <0.001^**^ |  |
|  | July to September | 69.5 (68.2, 70.7) | 57.4 (55.7, 59.1) | <0.001^**^ |  |
|  | October to December | 70.0 (68.6, 71.3) | 59.4 (57.7, 61.1) | <0.001^**^ |  |
|  | * Bivariate analysis of X-to-P conversion rate by Intervention Status using generalized estimating equations (standard errors adjusted for clustering by block/Intervention Status).  ** Statistically significant difference (p<0.05) in multivariate analysis of ‘X-to-P conversion rate by Intervention Status controlling for District, Place of residence and Time of year, using generalized estimating equations (standard errors adjusted for clustering by block/Intervention Status).  ^ns^ Statistically insignificant difference (p>0.05) between intervention and non-intervention areas, in the multivariate analysis | | | |  |

**Appendix Table 3e. Mean rate of remaining ‘X’ houses at the end of house-to-house activities of SIAs by district, place of residence, time of year and intervention status, March 2012 to September 2017**

|  | **By:** | **Percentage of remaining ‘X’ houses at the end of house-to-house activities of SIAs**  **(95% Confidence Interval)** | |  |  |
| --- | --- | --- | --- | --- | --- |
|  |  | **Intervention Areas**  (n [blocks]= 56;  Obs. per block=27**)** | **Non-intervention Areas**  (n [blocks]= 56;  Obs. per block=27**)** | **p-value** |  |
|  | **Overall** | 4.9 (4.8, 5.1) | 5.9 (5.8, 6.0) | <0.015^*^,^**^ |  |
|  |  |  |  |  |  |
|  | **District** |  |  |  |  |
|  | Baghpat | 6.7 (6.3, 7.0) | 6.6 (6.2, 6.9) | 0.891^ns^ |  |
|  | Bareilly | 5.3 (4.9, 5.7) | 6.2 (5.9, 6.4) | 0.500^ns^ |  |
|  | Mau | 2.8 (2.5, 3.1) | 5.8 (5.5, 6.2) | <0.001^*^ |  |
|  | Meerut | 4.6 (4.4, 4.9) | 4.6 (4.4, 4.9) | 0.976^ns^ |  |
|  | Moradabad | 3.5 (3.3, 3.8) | 3.8 (3.5, 4.1) | 0.203^ns^ |  |
|  | Muzaffarnagar | 7.5 (7.1, 7.9) | 8.1 (7.6, 8.6) | 0.679^ns^ |  |
|  | Rampur | 2.8 (2.7, 3.0) | 4.1 (3.9, 4.3) | 0.011^**^ |  |
|  | Saharanpur | 3.2 (3.0, 3.5) | 4.0 (3.6, 4.3) | 0.020^**^ |  |
|  | Sambhal | 5.6 (5.3, 5.8) | 7.2 (6.8, 7.5) | 0.054^ns^ |  |
|  | Shahjahanpur | 6.4 (6.0, 6.8) | 7.3 (6.9, 7.6) | 0.492^ns^ |  |
|  | Shamli | 6.4 (6.0, 6.8) | 7.4 (6.7, 8.0) | 0.094^ns^ |  |
|  | Sitapur | 4.0 (3.9, 4.1) | 5.7 (5.5, 5.9) | 0.002^**^ |  |
|  |  |  |  |  |  |
|  | **Place of residence** |  |  |  |  |
|  | Rural | 5.2 (5.1, 5.3) | 6.2 (6.0, 6.3) | 0.001^**^ |  |
|  | Urban | 2.9 (2.7, 3.1) | 3.7 (3.4, 3.9) | 0.001^**^ |  |
|  |  |  |  |  |  |
|  | **Time of year** |  |  |  |  |
|  | January to March | 4.9 (4.7, 5.1) | 5.9 (5.7, 6.1) | 0.001^**^ |  |
|  | April to June | 5.5 (5.3, 5.7) | 6.6 (6.4, 6.8) | <0.001^**^ |  |
|  | July to September | 4.2 (4.0, 4.4) | 4.9 (4.7, 5.1) | 0.001^**^ |  |
|  | October to December | 4.8 (4.5, 5.1) | 5.6 (5.4, 5.9) | 0.001^**^ |  |
|  | * Bivariate analysis of the rate of remaining ‘X’ houses at the end of SIAs by Intervention Status using generalized estimating equations (standard errors adjusted for clustering by block/Intervention Status).  ** Statistically significant difference (p<0.05) in multivariate analysis of the rate of remaining ‘X’ houses at the end of SIAs by Intervention Status controlling for District, Place of residence and Time of year, using generalized estimating equations (standard errors adjusted for clustering by block/Intervention Status).  ^ns^ Statistically insignificant difference (p>0.05) between intervention and non-intervention areas, in the multivariate analysis | | | |  |

**Appendix Table 3f. Mean refusal rate (resistant households per 10,000 visited households) at the beginning of SIAs by district, place of residence, time of year and intervention status, October 2012 to September 2017**

|  | **By:** | **Refusal rates at the beginning of SIAs**  **(95% Confidence Interval)** | |  |  |
| --- | --- | --- | --- | --- | --- |
|  |  | **Intervention Areas**  (n [blocks]= 56;  Obs. per block=22**)** | **Non-intervention Areas**  (n [blocks]= 56;  Obs. per block=22**)** | **p-value** |  |
|  | **Overall** | 8.0 (7.1, 8.8) | 3.3 (3.0, 3.6) | <0.003^*^,^**^ |  |
|  |  |  |  |  |  |
|  | **District** |  |  |  |  |
|  | Baghpat | 2.7 (2.1, 3.2) | 0.5 (0.3, 0.6) | <0.001^**^ |  |
|  | Bareilly | 5.0 (4.0, 6.1) | 2.0 (1.1, 2.0) | 0.153^ns^ |  |
|  | Mau | 26.7 (20.3, 33.2) | 5.4 (4.6, 6.1) | 0.081^ns^ |  |
|  | Meerut | 4.5 (3.0, 6.0) | 0.8 (0.7, 1.0) | <0.001^**^ |  |
|  | Moradabad | 16.6 (13.4, 19.8) | 11.5 (9.3, 13.7) | 0.070^ns^ |  |
|  | Muzaffarnagar | 5.5 (3.6, 7,4) | 1.3 (0.9, 1.7) | <0.001^**^ |  |
|  | Rampur | 6.5 (5.5, 7.4) | 3.5 (3.0, 4.0) | 0.016^**^ |  |
|  | Saharanpur | 3.1 (2.3, 4.0) | 1.3 (1.0, 1.6) | 0.078^ns^ |  |
|  | Sambhal | 17.7 (12.9, 22.5) | 7.6 (5.9, 9.4) | 0.003^**^ |  |
|  | Shahjahanpur | 4.9 (4.2, 5.6) | 3.4 (2.7, 4.0) | 0.214^ns^ |  |
|  | Shamli | 1.5 (1.0, 2.0) | 0.8 (0.4, 1.1) | 0.265^ns^ |  |
|  | Sitapur | 1.8 (1.5, 2.2) | 1.3 (1.1, 1.4) | 0.347^ns^ |  |
|  |  |  |  |  |  |
|  | **Place of residence** |  |  |  |  |
|  | Rural | 7.0 (6.1, 7.9) | 2.3 (2.1, 2.5) | <0.001^**^ |  |
|  | Urban | 16.2 (14.1, 18.2) | 11.4 (9.3, 13.5) | 0.039^**^ |  |
|  |  |  |  |  |  |
|  | **Time of year** |  |  |  |  |
|  | January to March | 7.6 (6.4, 8.7) | 3.3 (2.8, 3.8) | <0.001^**^ |  |
|  | April to June | 8.8 (7.1, 10.4) | 3.4 (2.8, 4.0) | <0.001^**^ |  |
|  | July to September | 6.2 (5.0, 7.5) | 2.9 (2.1, 3.7) | <0.001^**^ |  |
|  | October to December | 9.4 (6.6, 12.3) | 3.7 (2.8, 4.6) | 0.001^**^ |  |
|  | * Bivariate analysis of refusal rates at the beginning of SIAs by Intervention Status using generalized estimating equations (standard errors adjusted for clustering by block/Intervention Status).  ** Statistically significant difference (p<0.05) in multivariate analysis of refusal rates at the beginning of SIAs by Intervention Status controlling for District, Place of residence and Time of year, using generalized estimating equations (standard errors adjusted for clustering by block/Intervention Status).  ^ns^ Statistically insignificant difference (p>0.05) between intervention and non-intervention areas, in the multivariate analysis | | | |  |

**Appendix Table 3g. Mean Refusal-to-Acceptor conversion rate of SIAs by district, place of residence, time of year and intervention status, October 2012 to September 2017**

|  | **By:** | **Refusal-to-Acceptor conversion rates of SIAs**  **(95% Confidence Interval)** | |  |  |
| --- | --- | --- | --- | --- | --- |
|  |  | **Intervention Areas**  (n [blocks]= 56;  Obs. per block=22**)** | **Non-intervention Areas**  (n [blocks]= 56;  Obs. per block=22**)** | **p-value** |  |
|  | **Overall** | 73.7 (71.8, 75.5) | 65.5 (63.6, 67.3) | <0.004^*^,^**^ |  |
|  |  |  |  |  |  |
|  | **District** |  |  |  |  |
|  | Baghpat | 84.3 (78.4, 90.2) | 79.6 (72.5, 86.7) | 0.796^ns^ |  |
|  | Bareilly | 75.2 (68.7, 81.7) | 58.3 (51.1, 65.4) | 0.266^ns^ |  |
|  | Mau | 74.7 (69.4, 80.0) | 72.4 (67.4, 77.5) | 0.844^ns^ |  |
|  | Meerut | 65.5 (58.0, 72.9) | 61.8 (53.3, 70.4) | 0.755^ns^ |  |
|  | Moradabad | 64.6 (61.1, 68.2) | 57.8 (53.6, 62.0) | 0.121^ns^ |  |
|  | Muzaffarnagar | 74.8 (68.4, 81.3) | 69.4 (62.2, 76.7) | 0.630^ns^ |  |
|  | Rampur | 81.9 (76.7, 87.0) | 57.9 (52.8, 63.0) | 0.011^**^ |  |
|  | Saharanpur | 46.6 (35.9, 57.3) | 47.7 (39.8, 55.6) | 0.728^ns^ |  |
|  | Sambhal | 78.1 (74.8, 81.4) | 67.4 (63.3, 71.5) | 0.005^**^ |  |
|  | Shahjahanpur | 85.0 (80.6, 89.5) | 72.8 (67.5, 78.1) | 0.001^**^ |  |
|  | Shamli | 68.8 (53.5, 84.2) | 65.1 (54.6, 75.5) | 0.935^ns^ |  |
|  | Sitapur | 69.5 (61.5, 77.5) | 69.8 (64.5, 75.1) | 0.668^ns^ |  |
|  |  |  |  |  |  |
|  | **Place of residence** |  |  |  |  |
|  | Rural | 75.5 (73.4, 77.5) | 66.8 (64.8, 68.7) | 0.005^**^ |  |
|  | Urban | 61.8 (57.6, 65.9) | 55.9 (51.2, 60.6) | 0.175^ns^ |  |
|  |  |  |  |  |  |
|  | **Time of year** |  |  |  |  |
|  | January to March | 73.0 (69.9, 76.0) | 65.5 (62.6, 68.4) | 0.052^* a^ |  |
|  | April to June | 75.1 (71.7, 78.6) | 65.9 (62.4, 69.4) | 0.009^* a^ |  |
|  | July to September | 72.7 (68.2, 77.3) | 64.0 (59.3, 68.6) | 0.042^* a^ |  |
|  | October to December | 74.0 (69.7, 78.3) | 66.3 (62.0, 70.6) | 0.061^* a^ |  |
|  | * Bivariate analysis of Refusal-to-Acceptor conversion rates of SIAs by Intervention Status using generalized estimating equations (standard errors adjusted for clustering by block/Intervention Status).  ** Statistically significant difference (p<0.05) in multivariate analysis of Refusal-to-Acceptor conversion rates of SIAs by Intervention Status controlling for District and Place of residence, using generalized estimating equations (standard errors adjusted for clustering by block/Intervention Status).  ^ns^ Statistically insignificant difference (p>0.05) between intervention and non-intervention areas, in the multivariate analysis  a Not included in the multivariate GEE analyses, as the initial analysis (i.e., the model included: Refusal-to-Acceptor conversion rate, Intervention status and quarter of a year) found insignificant difference (p>0.05) among the Refusal-to-Acceptor conversion rates of quarters of a year. | | | |  |

**Appendix Table 3h. Mean refusal rate (resistant houses per 10,000 visited houses) at the end of SIAs by district, place of residence, time of year and intervention status, October 2012 to September 2017**

|  | **By:** | **Refusal rates at the end of SIAs**  **(95% Confidence Interval)** | |  |  |
| --- | --- | --- | --- | --- | --- |
|  |  | **Intervention Areas**  (n [blocks]= 56;  Obs. per block=22**)** | **Non-intervention Areas**  (n [blocks]= 56;  Obs. per block=22**)** | **p-value** |  |
|  | **Overall** | 1.9 (1.5, 2.2) | 1.2 (1.0, 1.3) | 0.093^ns^ |  |
|  |  |  |  |  |  |
|  | **District** |  |  |  |  |
|  | Baghpat | 0.3 (0.2, 0.4) | 0.1 (0.1, 0.1) | 0.012^*^ |  |
|  | Bareilly | 1.8 (1.3, 2.4) | 0.7 (0.5, 0.9) | 0.408^ns^ |  |
|  | Mau | 3.3 (2.4, 4.2) | 1.3 (1.0, 1.7) | 0.083^ns^ |  |
|  | Meerut | 1.3 (0.9, 1.7) | 0.3 (0.2, 0.4) | <0.001^*^ |  |
|  | Moradabad | 5.4 (4.5, 6.4) | 5.1 (4.0, 6.3) | 0.865^ns^ |  |
|  | Muzaffarnagar | 0.9 (0.7, 1.2) | 0.3 (0.2, 0.5) | 0.020^*^ |  |
|  | Rampur | 1.1 (0.8, 1.4) | 1.2 (1.0, 1.4) | 0.713^ns^ |  |
|  | Saharanpur | 1.9 (1.3, 2.5) | 0.8 (0.6, 0.9) | 0.462^ns^ |  |
|  | Sambhal | 4.9 (1.5, 8.3) | 2.4 (1.7, 3.1) | 0.267^ns^ |  |
|  | Shahjahanpur | 0.7 (0.5, 0.9) | 1.0 (0.8, 1.2) | 0.445^ns^ |  |
|  | Shamli | 0.3 (0.1, 0.4) | 0.3 (0.2, 0.4) | 0.911^ns^ |  |
|  | Sitapur | 0.5 (0.4, 0.7) | 0.4 (0.3, 0.5) | 0.610^ns^ |  |
|  |  |  |  |  |  |
|  | **Place of residence** |  |  |  |  |
|  | Rural | 1.4 (1.1, 1.8) | 0.7 (0.7, 0.8) | 0.028^*^ |  |
|  | Urban | 5.8 (5.0, 6.5) | 4.9 (3.9, 5.9) | 0.643^ns^ |  |
|  |  |  |  |  |  |
|  | **Time of year** |  |  |  |  |
|  | January to March | 1.7 (1.4, 1.9) | 1.1 (0.9, 4.5) | 0.148^ns^ |  |
|  | April to June | 1.9 (1.5, 2.2) | 1.2 (1.0, 1.5) | 0.107^ns^ |  |
|  | July to September | 1.6 (1.1, 2.0) | 1.1 (0.7, 1.6) | 0.311^ns^ |  |
|  | October to December | 2.7 (0.9, 4.5) | 1.3 (0.9, 1.7) | 0.147^ns^ |  |
|  | * Statistically significant difference (p<0.05) in the bivariate analysis of Refusal rates at the end of SIAs by Intervention Status using generalized estimating equations (standard errors adjusted for clustering by block/Intervention Status).  ^ns^ Statistically insignificant difference (p>0.05) between intervention and non-intervention areas, in the bivariate analysis  Note – Refusal rates at the end of SIAs do not differ by intervention status at the bivariate level; hence the differences by district, place of residence and Time of year were not assessed through a multivariate analysis. | | | |  |

**Appendix Table 3i. Mean level of community engagement in polio SIAs by district, place of residence, time of year and intervention status, March 2012 to September 2017**

|  | **By:** | **Community Engagement Index (in %)**  **(95% Confidence Interval)** | |  |  |
| --- | --- | --- | --- | --- | --- |
|  |  | **Intervention Areas**  (n [blocks]= 56;  Obs. per block=27**)** | **Non-intervention Areas**  (n [blocks]= 56;  Obs. per block=27**)** | **p-value** |  |
|  | **Overall** | 89.0 (88.8, 89.2) | 70.8 (70.6, 71.1) | < 0.001^*^,^**^ |  |
|  |  |  |  |  |  |
|  | **District** |  |  |  |  |
|  | Baghpat | 88.5 (88.2, 88.8) | 70.6 (70.2, 71.0) | < 0.001^**^ |  |
|  | Bareilly | 89.8 (89.3, 90.3) | 71.9 (71.4, 72.3) | < 0.001^**^ |  |
|  | Mau | 92.8 (92.1, 93.5) | 68.3 (67.9, 68.7) | < 0.001^**^ |  |
|  | Meerut | 88.6 (86.1, 87.1) | 77.5 (76.7, 78.3) | < 0.001^**^ |  |
|  | Moradabad | 86.6 (86.1, 87.1) | 73.6 (72.9, 74.2) | < 0.001^**^ |  |
|  | Muzaffarnagar | 88.7 (87.9, 89.4) | 68.9 (68.4, 69.4) | < 0.001^**^ |  |
|  | Rampur | 90.3 (90.0, 90.7) | 70.9 (70.5, 71.4) | < 0.001^**^ |  |
|  | Saharanpur | 90.6 (89.9, 91.3) | 75.4 (74.7, 75.6) | < 0.001^**^ |  |
|  | Sambhal | 85.3 (84.9, 85.7) | 74.9 (74.2, 75.6) | < 0.001^**^ |  |
|  | Shahjahanpur | 90.4 (89.9, 91.3) | 68.9 (68.2, 69.6) | < 0.001^**^ |  |
|  | Shamli | 88.2 (87.5, 88.9) | 70.2 (69.5, 70.9) | < 0.001^**^ |  |
|  | Sitapur | 89.0 (88.5, 89.4) | 60.9 (60.5, 61.4) | < 0.001^**^ |  |
|  |  |  |  |  |  |
|  | **Place of residence** |  |  |  |  |
|  | Rural | 89.3 (89.1, 89.4) | 70.6 (70.3, 70.9) | < 0.001^* a^ |  |
|  | Urban | 86.7 (86.1, 87.2) | 72.9 (72.2, 73.7) | < 0.001^* a^ |  |
|  |  |  |  |  |  |
|  | **Time of year** |  |  |  |  |
|  | January to March | 89.3 (89.1, 89.6) | 71.3 (70.8, 71.8) | < 0.001^**^ |  |
|  | April to June | 88.4 (88.1, 88.8) | 70.4 (69.9, 70.9) | < 0.001^**^ |  |
|  | July to September | 89.5 (89.0, 89.9) | 70.4 (69.8, 71.0) | < 0.001^**^ |  |
|  | October to December | 88.6 (88.2, 89.1) | 71.1 (70.3, 71.8) | < 0.001^**^ |  |
|  | * Bivariate analysis of Community Engagement Index by Intervention Status using generalized estimating equations (standard errors adjusted for clustering by block/Intervention Status)  ** Statistically significant difference (p<0.01) in multivariate analysis of Community Engagement Index by Intervention Status controlling for District, Time of year, and including interaction of District with Intervention Status, using generalized estimating equations (standard errors adjusted for clustering by block/Intervention Status).  a Not included in the multivariate GEE analyses, as the initial analysis (i.e., the model included: Community Engagement Index, Intervention status and Place of residence) found an insignificant difference (p=0.684) between the Community Engagement Index of rural and urban areas. | | | |  |

**Appendix Table 4. Linear post-intervention (post-polio-endemic period) trend of polio SIA outcomes by intervention status**

|  | **Outcome indicator** | **Area** | **Coefficient** | **Std. Err.*** | **t stat.** | **p value** | **95 % Confidence Interval** | |  |
| --- | --- | --- | --- | --- | --- | --- | --- | --- | --- |
|  | SIA coverage | Intervention | -0.0010 | 0.0210 | -0.05 | 0.964 | -0.0420 | 0.0401 |  |
|  |  | Non-intervention^a^ | 0.0214 | 0.0284 | 0.75 | 0.451 | -0.0343 | 0.0771 |  |
|  |  | Difference^†^ | -0.0224 | 0.0353 | -0.63 | 0.526 | -0.0916 | 0.0469 |  |
|  | Booth coverage | Intervention | 0.2471 | 0.0451 | 5.48 | <0.001 | 0.1580 | 0.3361 |  |
|  |  | Non-intervention^b^ | -0.1385 | 0.1114 | -1.24 | 0.216 | -0.3587 | 0.0817 |  |
|  |  | Difference^†^ | 0.3856 | 0.1202 | 3.21 | 0.002 | 0.1480 | 0.6231 |  |
|  | X-to-P conversion rate | Intervention | 0.0040 | 0.1069 | 0.04 | 0.970 | -0.2060 | 0.2141 |  |
|  |  | Non-intervention^c^ | -0.0934 | 0.1093 | -0.86 | 0.393 | -0.3082 | 0.1213 |  |
|  |  | Difference^†^ | 0.0975 | 0.1529 | 0.64 | 0.524 | -0.2029 | 0.3978 |  |
|  | Rate of remaining ‘X’ houses at the end of SIAs | Intervention | -0.0612 | 0.0161 | -3.81 | <0.001 | -0.0928 | -0.0296 |  |
|  |  | Non-intervention^d^ | -0.0455 | 0.0136 | -3.35 | <0.001 | -0.0721 | -0.0188 |  |
|  |  | Difference^†^ | -0.0157 | 0.0210 | -0.75 | 0.456 | -0.0571 | 0.0256 |  |
|  | Refusal-to-Acceptor conversion rate | Intervention | 0.0647 | 0.3166 | 0.20 | 0.839 | -0.5574 | 0.6867 |  |
|  |  | Non-intervention^e^ | 0.1593 | 0.4125 | 0.39 | 0.700 | -0.6511 | 0.9698 |  |
|  |  | Difference^†^ | -0.0946 | 0.5200 | -0.18 | 0.856 | -1.1163 | 0.9270 |  |
|  | Refusal rate at the end of SIAs | Intervention | -0.0838 | 0.0111 | -7.53 | <0.001 | -0.1058 | -0.0619 |  |
|  |  | Non-intervention^f^ | 0.0008 | 0.0729 | 0.01 | 0.991 | -0.1430 | 0.1446 |  |
|  |  | Difference^†^ | -0.0847 | 0.0738 | -1.15 | 0.252 | -0.2301 | 0.0608 |  |
|  | Community Engagement Index of polio SIAs | Intervention | 0.1567 | 0.0245 | 6.39 | <0.001 | 0.1083 | 0.2051 |  |
|  |  | Non-intervention^g^ | -0.0325 | 0.0644 | -0.50 | 0.614 | -0.1598 | 0.0947 |  |
|  |  | Difference^†^ | 0.1892 | 0.0689 | 2.75 | 0.007 | 0.0530 | 0.3254 |  |
|  | * Semi-robust standard error † Difference between intervention and non-intervention areas  ITSA analysis included 56 intervention (CMC) areas and selected:  ^a^ 40 non-intervention areas; ^b^ 2 non-intervention areas; ^c^ 8 non-intervention areas; ^d^ 11 non-intervention areas;  ^e^ 21 non-intervention areas; ^f^ 3 non-intervention areas; ^g^ 2 non-intervention areas | | | | | | | |  |
