# Supplementary material for: Effectiveness of a community-level social mobilization intervention in achieving the outcomes of polio vaccination campaigns during the post-polio-endemic period: Evidence from CORE Group polio project in Uttar Pradesh, India
Source: BMC Public Health. 2021 Jul 10;21:1371. doi: 10.1186/s12889-021-11425-0 (PMC8272292; doi:10.1186/s12889-021-11425-0)
Supplement: Supplementary file 2 — Additional file 2: Fig. S1. Trends in actual and predicted values of seven indicators of intervention and selected non-intervention area, using two-group interrupted time-series analysis. [file 12889_2021_11425_MOESM2_ESM.docx]

**Appendix Figures**


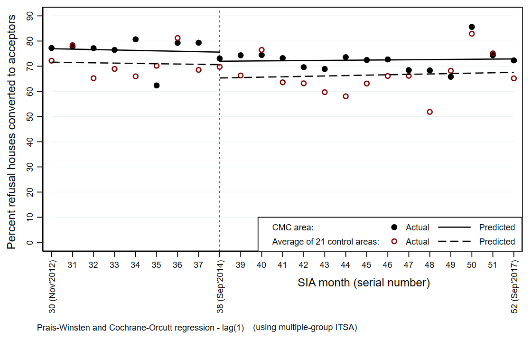


**E. Refusal-to-Acceptor conversion rate of SIAs**


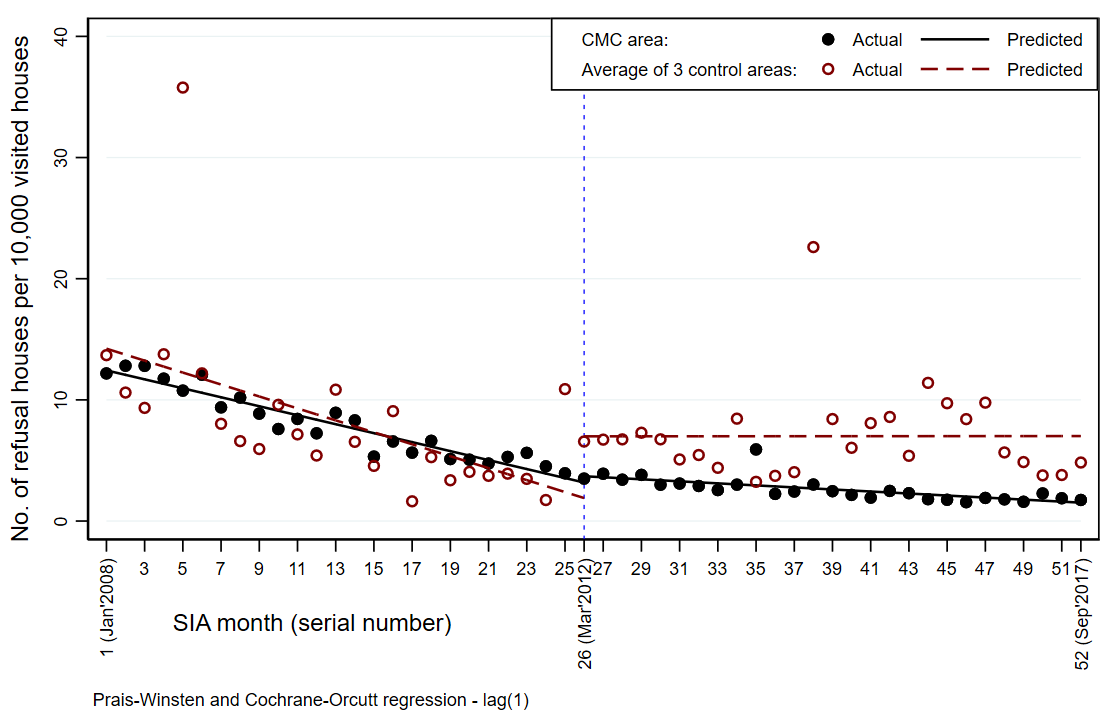


**F. Refusal rate at the end of SIAs**


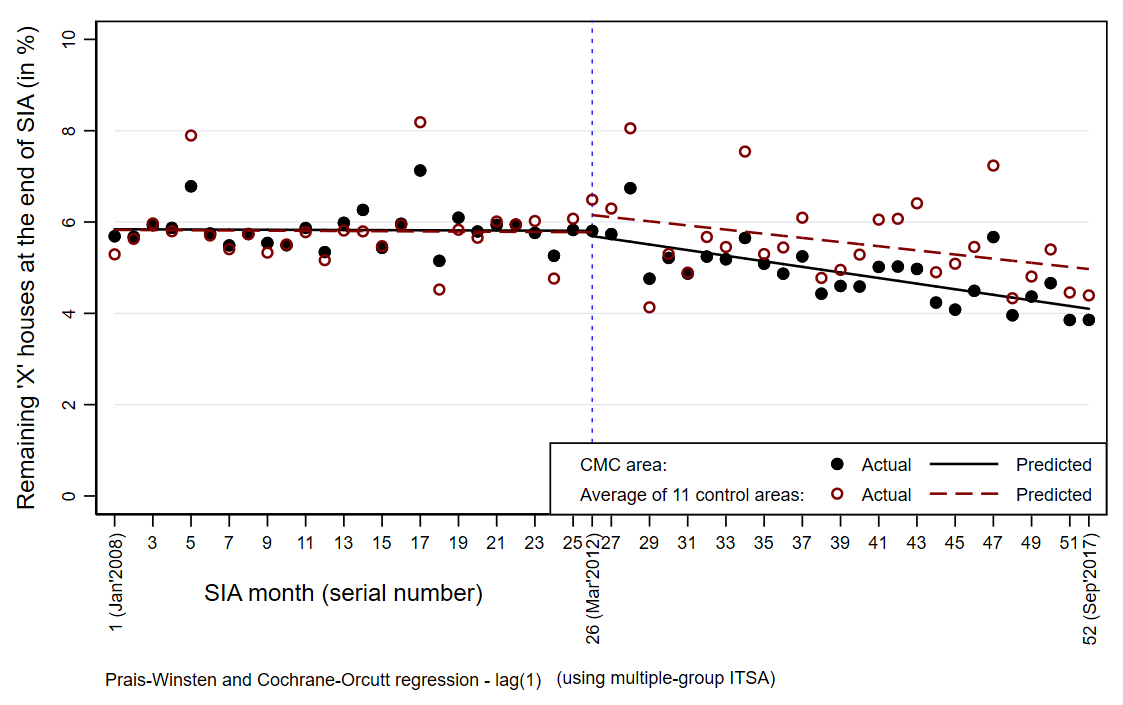


**D. Rate of remaining ‘X’ houses at the end of SIAs**


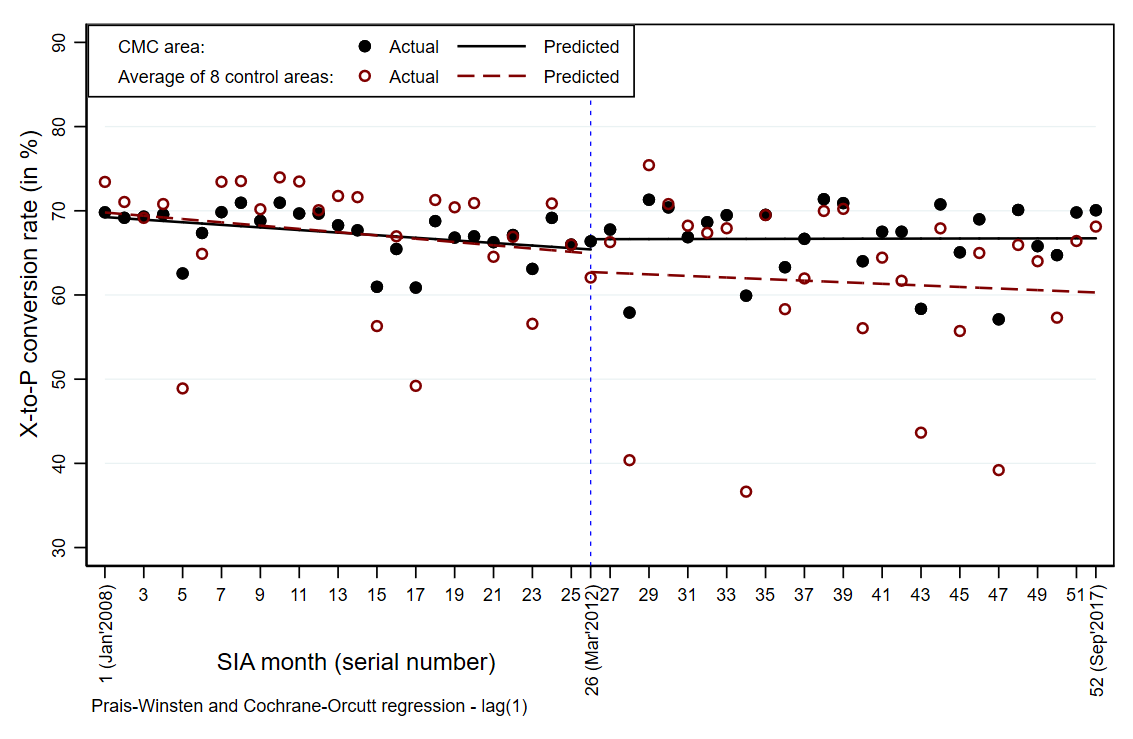


**C. X-to-P conversion rate**


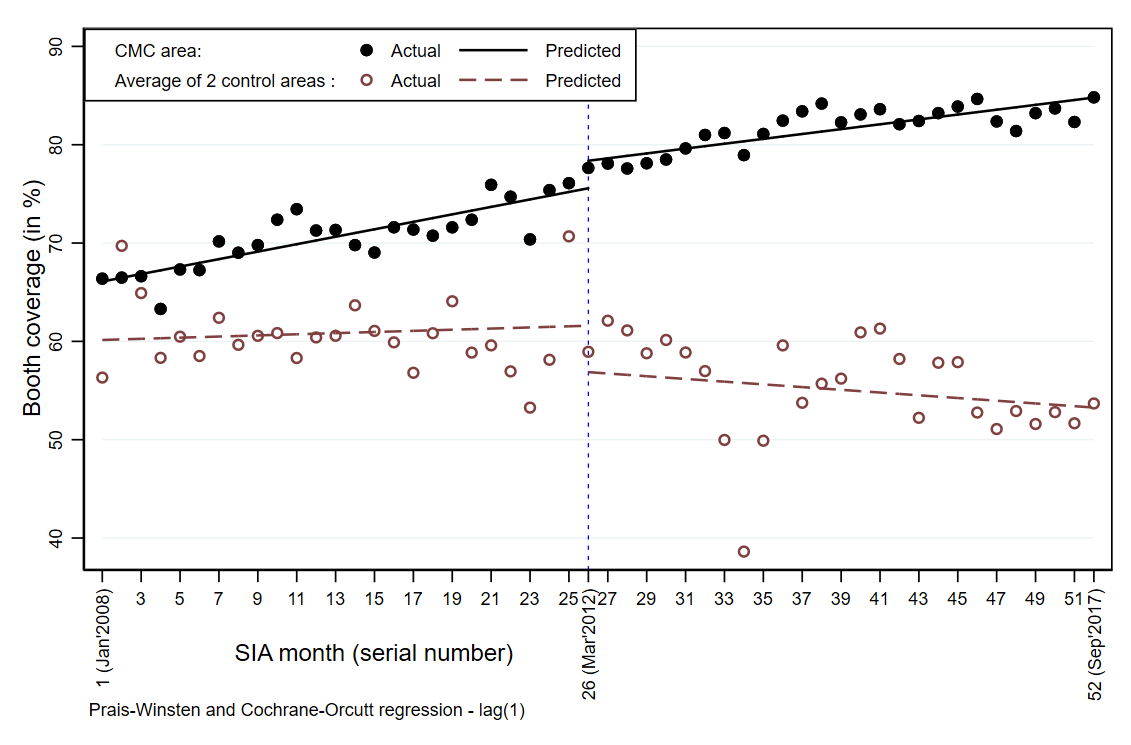


**B. Booth coverage**


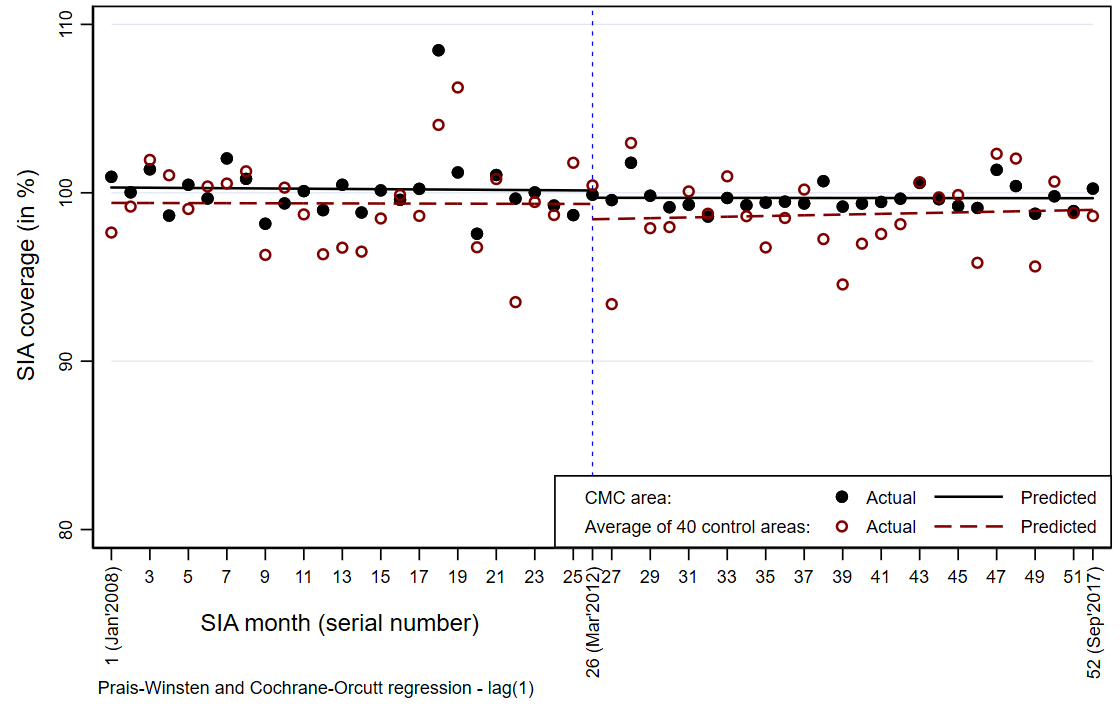


**A. SIA coverage**

**Appendix Figure 1. Trends in actual and predicted values of seven indicators of intervention and selected non-intervention area**, **using two-group interrupted time-series analysis.** the vertical broken blue line divides the entire study period into two periods, i.e. 1) polio-endemic period and 2) post-polio-endemic period. Each circle represents the mean value for an indicator. The Black circles and black line represent intervention areas and brown hallow circles and broken brown line represent non-intervention areas.


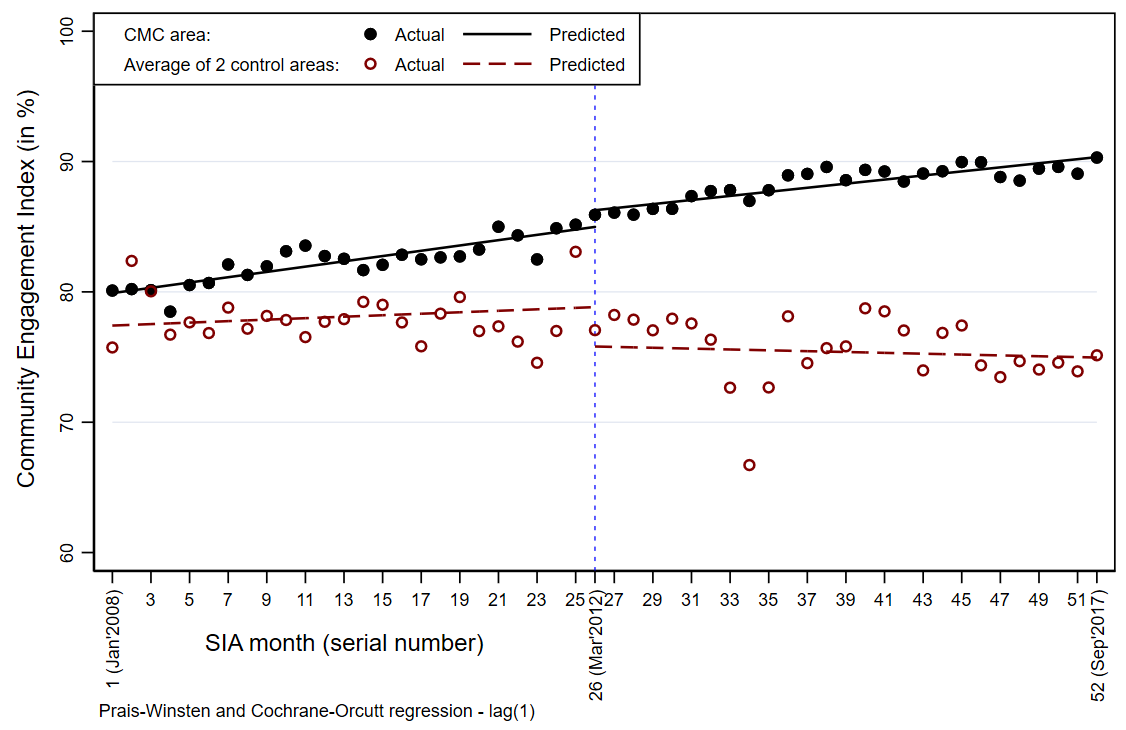


**G. Community Engagement Index of SIAs**
